# Phenotypic and Genotypic Characterization of *Acinetobacter* spp. Panel Strains: A Cornerstone to Facilitate Antimicrobial Development

**DOI:** 10.3389/fmicb.2019.00559

**Published:** 2019-03-26

**Authors:** Roshan D'Souza, Naina A. Pinto, Nguyen Le Phuong, Paul G. Higgins, Thao Nguyen Vu, Jung-Hyun Byun, Young Lag Cho, Jong Rak Choi, Dongeun Yong

**Affiliations:** ^1^Department of Laboratory Medicine, Research Institute of Bacterial Resistance, Yonsei University College of Medicine, Seoul, South Korea; ^2^J. Craig Venter Institute, Rockville, MD, United States; ^3^Brain Korea 21 PLUS Project for Medical Science, Yonsei University, Seoul, South Korea; ^4^Institute for Medical Microbiology, Immunology and Hygiene, University of Cologne, Cologne, Germany; ^5^German Centre for Infection Research, Partner site Bonn-Cologne, Germany; ^6^Department of Laboratory Medicine, Gyeongsang National University College of Medicine, Jinju, South Korea

**Keywords:** *Acinetobacter*, panel strains, antimicrobial resistance, whole-genome sequencing, phenotypic characterization

## Abstract

*Acinetobacter* spp. have emerged as significant pathogens causing nosocomial infections. Treatment of these pathogens has become a major challenge to clinicians worldwide, due to their increasing tendency to antibiotic resistance. To address this, much revenue and technology are currently being dedicated toward developing novel drugs and antibiotic combinations to combat antimicrobial resistance. To address this issue, we have constructed a panel of *Acinetobacter* spp. strains expressing different antimicrobial resistance determinants such as narrow spectrum β-lactamases, extended-spectrum β-lactamases, OXA-type-carbapenemase, metallo-beta-lactamase, and over-expressed AmpC β-lactamase. Bacterial strains exhibiting different resistance phenotypes were collected between 2008 and 2013 from Severance Hospital, Seoul. Antimicrobial susceptibility was determined according to the CLSI guidelines using agar dilution method. Selected strains were sequenced using Ion Torrent PGM system, annotated using RAST server and analyzed using Geneious pro 8.0. Genotypic determinants, such as acquired resistance genes, changes in the expression of efflux pumps, mutations, and porin alternations, contributing to the relevant expressed phenotype were characterized. Isolates expressing ESBL phenotype consisted of *bla*_PER−1_ gene, the overproduction of intrinsic AmpC beta-lactamase associated with IS*Aba1* insertion, and carbapenem resistance associated with production of carbapenem-hydrolyzing Ambler class D β-lactamases, such as OXA-23, OXA-66, OXA-120, OXA-500, and metallo-β-lactamase, SIM-1. We have analyzed the relative expression of Ade efflux systems, and determined the sequences of their regulators to correlate with phenotypic resistance. Quinolone resistance-determining regions were analyzed to understand fluoroquinolone-resistance. Virulence factors responsible for pathogenesis were also identified. Due to several mutations, acquisition of multiple resistance genes and transposon insertion, phenotypic resistance decision scheme for for evaluating the resistance proved inaccurate, which highlights the urgent need for modification to this scheme. This complete illustration of mechanism contributing to specific resistance phenotypes can be used as a target for novel drug development. It can also be used as a reference strain in the clinical laboratory and for the evaluation of antibiotic efficacy for specific resistance mechanisms.

## Introduction

*Acinetobacter* spp. are non-motile, non-fermenting Gram-negative bacteria. Over the years, several species have been identified, and the most common and clinically significant are *Acinetobacter baumannii, Acinetobacter pittii*, and *Acinetobacter nosocomialis* (Chen et al., 2014). These bacteria have emerged as the most troublesome pathogens in hospital settings, due to their rapid colonization and infection. Incidence and mortality due to *A. nosocomialis* and *A. pittii* are lower than those due to *A. baumannii*; however, these are frequently isolated from nosocomial infections (Wisplinghoff et al., 2012). *Acinetobacter* spp. have been implicated in many pathological conditions such as ventilator-associated pneumonia, urinary tract infections, skin and wound infections, infective endocarditis, bacteremia, and secondary meningitis (Fishbain and Peleg, 2010; Garnacho-Montero and Amaya-Villar, 2010; Visca et al., 2011; Chusri et al., 2014). These infections have become challenging to treat due to their widespread multidrug resistance owing to mechanisms such as horizontal gene transfer, increased expression of β-lactamases, alterations of membrane permeability, and increased expression of efflux pumps (Singh et al., 2013); (Blair et al., 2015).

For several decades, numerous research have been conducted to understand the mechanisms of resistance and to control its dissemination in clinical settings. Considering the severity of infections, we have constructed a series of panel strains of *Acinetobacter* spp. expressing different resistance phenotypes such as narrow spectrum β-lactamase and oxacillinase, extended spectrum β-lactamase (ESBL), OXA-type carbapenemase, metallo-β-lactamase (MBL), and over-expressing AmpC β-lactamase. These strains were characterized genotypically using massive parallel sequencing (MPS) technology to understand the observed phenotypes. In this study, we have performed detailed analysis of the whole genome sequence (WGS) related to multidrug-resistance mechanisms, such as acquisition of β-lactamases, transposon insertions, mutations in porins, and changes in efflux pumps, and interpreted the discrepancy observed in phenotypic changes to relevant antibiotics. These panel strains can be used in hospital settings as reference strains, and also in the pharmaceutical industry to check the efficacy of new antibiotic drugs on pathogens expressing different resistance determinants. These strains can be distributed world-wide to institutions working on discovery of novel antibiotics, aiding in their characterization.

## Materials and Methods

### Bacterial Strains

All bacterial strains were collected from Severance Hospital, Seoul from 2008 to 2013. Almost 4,000 strains were shortlisted depending on their *in-silico* resistance prediction from the hospital patient database, according to the resistance determination decision tree to interpret the type of resistance based on phenotypic observation by François et al. (2004) and Richard Bonnet (2010). Strains were categorized according to their resistance phenotype such as narrow spectrum β-lactamase and oxacillinase, ESBL, OXA-type-carbapenemase, MBL and over-expressed AmpC β-lactamase. Bacteria were identified using the direct colony method with MALDI-TOF MS (Bruker Daltonics, Bremen, Germany). In addition, RNA polymerase β-subunit gene (*rpoB*)-based identification was used to delineate species within the *Acinetobacter* genus (La Scola et al., 2006).

### Susceptibility Tests

Initially, disc diffusion assays were performed on Muller Hinton agar plates with antibiotic discs containing piperacillin, ampicillin, piperacillin-tazobactam, ceftazidime, cefepime, imipenem, meropenem, ciprofloxacin, ceftazidime-clavulanate, ampicillin-sulbactam, and aztreonam to detect antibiotic susceptibility. In addition, the minimum-inhibitory concentrations (MIC) for bacterial strains were determined using agar dilution technique. E-test was used to measure the MIC of levofloxacin, trimethoprim/sulfamethoxazole, tigecycline, tetracycline, gentamicin, rifampicin, clindamycin, erythromycin and chloramphenicol. All of the procedures and results interpretation followed the Clinical and Laboratory Standards Institute (CLSI) guidelines. AmpC β-lactamase-, MBL, and ESBL-producing strains were selected using ertapenem-amino phenylboronic acid (APBA), imipenem-EDTA, and cefepime-clavulanate double disk synergy tests, respectively (Lee et al., 2001). Modified Hodge tests were also performed with cefoxitin disk for AmpC beta-lactamase detection, and imipenem disk for carbapenemase detection (Lee et al., 2010).

### Whole Genome Sequencing and Bioinformatics Analysis

A few strains from each phenotypic resistance class were randomly selected and cultured overnight. Genomic DNA extractions were performed using Wizard genomic DNA purification kit (Promega, WI, USA) with a few modifications to the manufacturer's protocol, such as adding 5 μl of RNase solution during cell lysis as well as incubating the supernatant carrying the DNA at −20°C for 1 h after addition of isopropanol. DNA concentration was measured using Qubit dsDNA BR assay kit (Molecular Probes, OR, USA) before sequencing.

Whole genome libraries were prepared using Ion plus fragment library kit, and Emulsion PCR was carried out using Ion one touch 200 Template kit v2 DL (Life technologies, CA, USA). Sequencing of the amplicon libraries was carried out on a 318 chip, using Ion Personal Genome Machine Ion Torrent sequencer through Ion Sequencing 200 kit (Life technologies, CA, USA). The resultant reads were assembled using MIRA plug-in (version 4.0) of Torrent suite software. Genome assemblies were annotated using RAST annotation pipeline, and further validated with Geneious pro 8.0 (Aziz et al., 2008; Kearse et al., 2012). Genes encoding the efflux systems, porins, and virulence factors of the panel strains were aligned using Clustal Omega, and verified for the polymorphisms (Sievers et al., 2011). Resistance genes were identified using Resfinder (Zankari et al., 2012), and manually curated using NCBI BLAST. Multi-locus sequence typing was performed using MLST 1.8 (Zankari et al., 2013) and *Acinetobacter baumannii* MLST website (Jolley and Maiden, 2010).

### Outer Membrane Protein Detection

Bacterial cells were grown in Muller-Hinton broth until logarithmic phase, and centrifuged at 5,000 g for 15 min, washed twice in 10-mM phosphate buffer, and lysed by sonication at 18–20% amplitude for 2 × 30 s cycles, each comprised 6 × 5 s sonication steps separated by 1 s of no sonication, and 30 s of no sonication between the two cycles. Unbroken cells were eliminated using centrifugation at 3,000 g for 5 min, and outer membrane was solubilized with 2% sodium lauroyl sarcosinate. Insoluble outer membrane fraction was recovered by ultracentrifugation at 25,000 g for 1 h, as described previously (Hernandez-Alles et al., 1999). OMP profiles were determined using SDS-PAGE using Mini-Protean TGX gels (12%), followed by Coomassie blue staining (Bio-Rad, CA, USA). Additionally, OMPs were identified using Matrix-Assisted Laser Desorption-Time of Flight Mass Spectrometry on Tinkerbell LT (ASTA, Suwon, Korea), as described (Pinto et al., 2017). All experiments were repeated three times independently to check for reproducibility of the results.

### Quantitative Real-Time RT-qPCR

Total RNA of the 12 *Acinetobacter* spp. isolates were extracted from exponentially grown bacterial cells with optical density at 600 nm of 0.7–0.8, using RNeasy Mini Kit (Qiagen, Hilden, Germany). Quantity and quality of RNA samples were checked using NanoDrop spectrophotometer (ND- 2000 Thermo scientific, USA). RNA samples with 260/280 ratio from 1.9 to 2.1, 260/230 ratio from 2.0 to 2.5 were used for further analysis. All of the RNA samples were adjusted to the same concentration. Then, 1 μg of total RNA was used to synthesize cDNA by reverse transcription using M-MLV cDNA Synthesis Kit (Enzynomics, Korea) in a 20 μl reaction using 50 μM random hexamers. cDNA was further diluted and stored at −20°C until PCR. Real-time PCR was performed with a 20-μl reaction volume containing 2 μl (100ng) of cDNA, 1X iQ SYBR Green Supermix (Bio-Rad, CA, USA), and gene-specific primers, 300 nM each (for *adeB, adeG, adeJ, baeSR, carO, 33-36kDa omp*, and *oprD genes*), on StepOne Real-Time PCR System (Life technologies, CA, USA) with the following cycle: 1 cycle at 95°C for 3 min followed by 40 cycles of 95°C for 10 s and 56°C for 1 min. Dissociation curve was generated to check PCR amplification specificity. In each run, 2 μl RNase-free water was used as a no template control (NTC) for each gene. The primers used for RT-qPCR were designed using Primer3web (version 4.1.0) (Untergasser et al., 2012), validated using Geneious pro 8.0. (Kearse et al., 2012), synthesized commercially by Macrogen, Inc., Korea, and are shown in Table S9. Different primers were used for different species due to the polymorphism identified in efflux pumps. Each experiment was performed in triplicates at least twice independently. The changes in expression level for each gene was calculated according to a previous study (Livak and Schmittgen, 2001). In brief, for each sample, the threshold cycle (Ct) of target gene was determined and normalized to Ct value of *rpoB* gene, and then calculated relatively to the calibrator (strain YMC/2009/2/B2968) using formula 2^−ΔΔ*Ct*^ (data is represented as mean ± standard error). Detailed experimental conditions used in RT-qPCR based on MIQE requirements are described in Table S10.

## Results and Discussion

Among the 4,000 *Acinetobacter* spp. screened initially, we selected 26 isolates showing different phenotypic resistances, i.e., two ESBL-, six high-level AmpC β-lactamase-, ten OXA-type-carbapenemase-, five MBL-, two narrow-spectrum β-lactamase-, one narrow-spectrum oxacillinase-producing strains, in addition to a wild type strain, susceptible to all tested antibiotics (Table S1). Among these YMC2003/5/C86, YMC2003/1/R306 in ESBL's; YMC2009/2/B6756, YMC2012/7/R3167 among over-expressed AmpC beta-lactamase; YMC2011/2/C582, YMC2011/7/R812, YMC2012/1/R79, and YMC2012/9/R2209 in OXA-type-carbapenemase; YMC2013/3/R2081 in MBL; YMC2010/8/T346 in narrow spectrum beta-lactamase; and YMC2009/2/B2968 in narrow-spectrum oxacillinase were randomly picked and sequenced to further characterize the phenotypic and genotypic correlation (Tables 1, 2). The draft genome sequences of strains YMC2003/5/C86, YMC2003/1/R306, YMC2009/2/B6756, YMC2012/7/R3167, YMC2011/2/C582, YMC2011/7/R812, YMC2012/1/R79, YMC2012/9/R2209, YMC2013/3/R2081, YMC2010/8/T346, and YMC2009/2/B2968 have been deposited at DDBJ/ENA/GenBank under the accession MKHG00000000, MKHH00000000, MKHI00000000, MKHJ00000000, MKHK00000000, MKHL00000000, MKHM00000000, MKHN00000000, MKHO00000000, and MKHP00000000, respectively.

**Table 1:** Selected list of *Acinetobacter* spp. panel strains and its minimum inhibitory concentration with its sequence types.

**Strains**	**ST**	**SAM**	**PIP**	**PIP-TZ**	**CAZ**	**FEP**	**IPM**	**MEM**	**CAZ-CLV**	**TET**	**TGC**	**TS**	**RI**	**CM**	**EM**	**CL**	**GM**
**ESBL**			R	I/R	R	R	S	S									
*A. baumannii* YMC2003/5/C86	423	32, R	256, R	128, R	64, R	64, R	32, R	32, R	8, S	256, R	3	32	6	256	32	256	256, R
*A. nosocomialis* YMC2003/1/R306	948	32, R	256, R	32, I	64, R	64, R	2, S	4, S	4, S	24, R	0.19	4	4	128	12	16	64, R
**Over-expressed AmpC** **β-lactamase**			I/R	I/R	R	I/R	S	S									
*A. baumannii* YMC2009/2/B6756	191	32, R	256, R	256, R	256, R	32, R	2, S	4, S	64, R	64, R	1.5	32	4	256	256	256	256, R
*A. baumannii* YMC2012/7/R3167	208	32, R	256, R	8, S	64, R	32, R	1, S	4, S	64, R	256, R	1.5	1	6	256	12	256	4, S
**OXA-type carbapenemase**			R	R	S	S	I/R	I/R									
*A. baumannii* YMC2011/7/R812	1386	32, R	256, R	256, R	4, S	16, I	16, R	32, R	4, S	2, S	0.09	32	1.5	96	6	128	0.5, S
*A. baumannii* YMC2012/1/R79	191	32, R	256, R	256, R	256, R	128, R	32, R	64, R	64, R	256, R	1	32	3	256	256	256	256, R
*A. baumannii* YMC2011/2/C582	208	128, R	256, R	256, R	256, R	128, R	256, R	128, R	64, R	32, R	2	32	32	256	256	256	256, R
*A. baumannii* YMC2012/9/R2209	229	32, R	256, R	256, R	256, R	64, R	8, I	32, R	64, R	6, I	0.75	32	4	256	12	256	1.5, S
**Metallo-β-lactamase**			R	R	R	R	R	R									
*A. pittii* YMC2013/3/R2081	1030	16, R	256, R	4, S	256, R	64, R	4, S	64, R	32, R	32, R	0.38	32	32	192	256	128	256, R
**Narrow-spectrum** **β-lactamase**			I/R	I/R	S	S	S	S									
*A. pittii* YMC2010/8/T346	1385	4, S	32, I	0.5, S	4, S	4, S	2, S	16, R	4, S	4, S	0.19	0.38	3	96	32	64	1, S
**Narrow-spectrum oxacillinase**			R	R	S	S	S	S									
*A. pittii* YMC2009/2/B2968	1638	2, S	32, I	0.5, S	4, S	4, S	0.25, S	0.5, S	4, S	2, S	0.09	0.38	2	48	6	32	0.5, S
**Wild type**			S	S	S	S	S	S									
*A. baumannii* YMC2013/1/R3000		4, S	256, R	0.5, S	8, S	16, I	0.25, S	1, S	4, S	2, S	0.125	0.19	3	96	8	48	1.5, S

*R, Resistant; I, Intermediate; S, susceptible; SAM, Ampicillin/sulbactam; PIP, piperacillin; PIP/TZ, piperacillin-tazobactam; CAZ, ceftazidime; FEP, cefepime; IPM, imipenem; MEM, meropenem; CAZ/CLV, ceftazidime-clavulanate, TET, Tetracycline; TGC, Tigecycline; TS, Trimethoprim sulfamethoxazole; RI, Rifampicin; CM, Clindamycin; EM, Erythromycin; CL, Chloramphenicol; GM, Gentamicin*.

**Table 2:** Resistome analysis of the *Acinetobacter* spp. strains representing β-Lactamases and Aminoglycoside-modifying enzymes.

	**β-Lactamase**																		**Aminoglycoside-modifying enzyme**								
				**ADC's**																							
	**CARB-8**	**PER-1**	**TEM-1D**	**ADC-22**	**ADC-25**	**ADC-30**	**ADC-31**	**ADC-41**	**ADC-77**	**OXA-82**	**OXA-66**	**OXA-23**	**OXA-120**	**OXA-421**	**OXA-499**	**OXA-213**	**OXA-500**	**SIM-1**	**Aac(3')-Ia**	**Aac(6')-Il**	**Aph(3')-Ic**	**aadA1/24**	**aacA**	**aadB**	**armA**	**strA**	**strB**
**ESBL**																											
YMC2003/5/C86		•	•				•			•									•	•	•					•	•
YMC2003/1/R306		•			•																			•		•	•
**OVER-EXPRESSED AMPC** **β-LACTAMASE**																											
YMC2009/2/B6756			•			•					•								•			•	•		•		
YMC2012/7/R3167						•					•															•	•
**OXA-TYPE CARBAPENEMASES**																											
YMC2011/7/R812									•			•	•														
YMC2012/1/R79			•			•					•	•							•			•			•		
YMC2011/2/C582		•				•					•	•									•	•			•	•	•
YMC2012/9/R2209						•				•																•	•
**METALLO-β-LACTAMASE**																											
YMC2013/3/R2081	•	•			•												•	•				•					
**NARROW-SPECTRUM** **β-LACTAMASE**																											
YMC2010/8/T346								•							•	•											
**NARROW-SPECTRUM OXACILLINASE**																											
YMC2009/2/B2968				•										•													

### Extended-Spectrum Beta-Lactamases

In Korea a high prevalence of *bla*_PER−1_ ESBL-producing *Acinetobacter* spp. was reported between 2001 and 2005 (Yong et al., 2003), and the level has been reducing over the years. The *bla*_PER−1_ belongs to class A extended-spectrum beta-lactamase, which has been detected in *P. aeruginosa* (Ranellou et al., 2012), *P. mirabilis* (Pagani et al., 2004), *S. enterica* (Poirel et al., 2005), and *Acinetobacter* spp. (Naas et al., 2006), and disseminated worldwide since its first detection in France on 1993 (Nordmann et al., 1993). ESBLs are a class of group A β-lactamases, which hydrolyze third generation cephalosporin's but are inhibited by beta-lactamase inhibitors like clavulanic acid (Bradford, 2001; Jacoby and Munoz-Price, 2005). Antimicrobial susceptibility for beta-lactams is similar in ESBLs and high-level AmpC β-lactamase-producing *Acinetobacter* spp. We have categorized the strains depending according to the presence of ESBL or AmpC-producing genes, along with IS elements.

**Figure 1:**
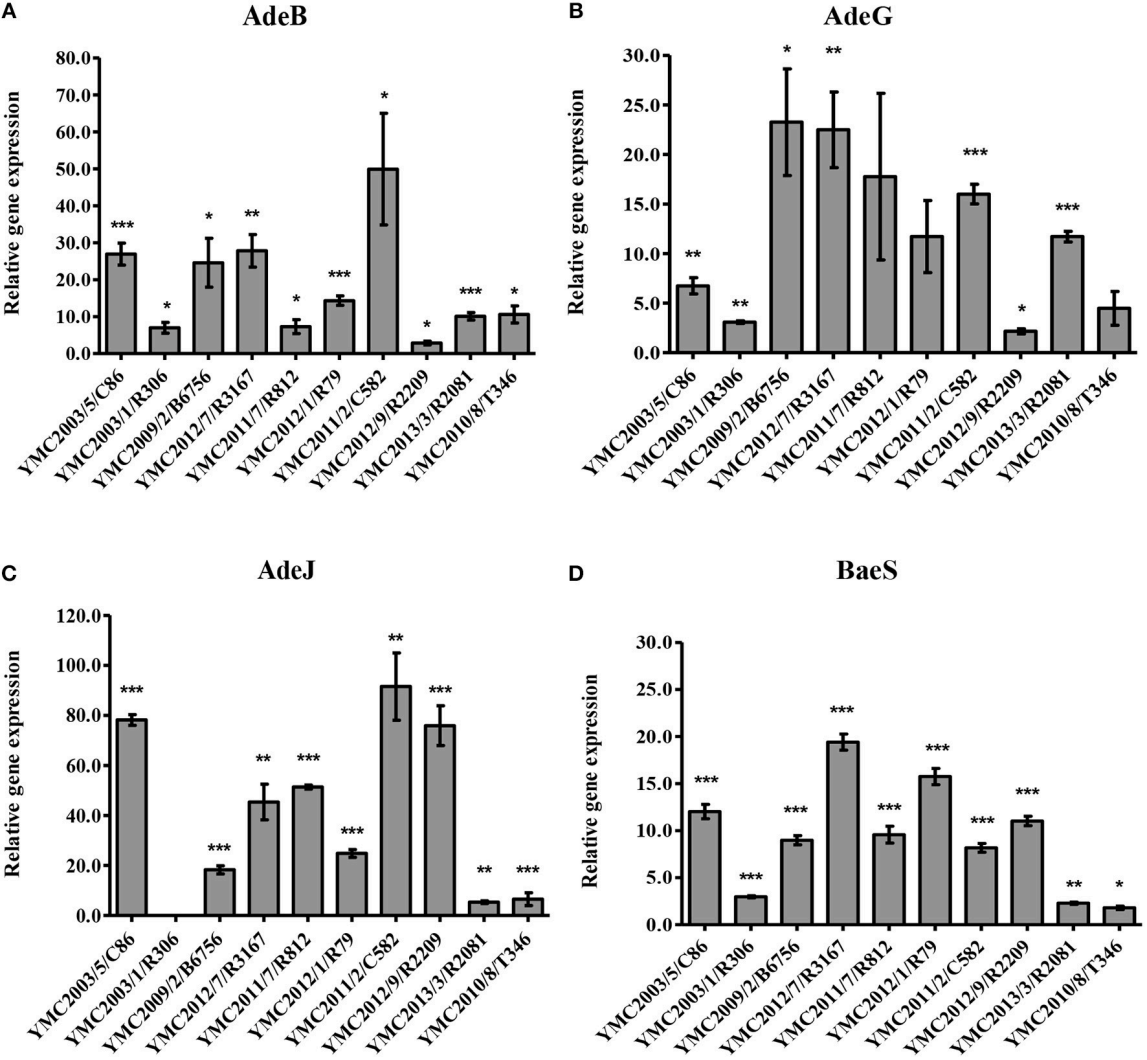
Expression of **(A)** AdeB, **(B)** AdeG, **(C)** AdeJ, and **(D)** BaeS relative to rpoB. Susceptible strain *A. pittii* YMC2009/2/B2968 was used as a reference and each isolate was tested in triplicate in two independent experiments. The data represent the mean ± standard error for three independent replicates. The significant difference of expression levels between samples were indicated by bars and asterisks as follows: ^*^p < 0.05, ^**^p < 0.01, and ^***^p < 0.001 using the Student's *t*-test.

- *Acinetobacter baumannii* YMC2003/5/C86: This strain was resistant to all antibiotics tested in this study, except ceftazidime-clavulanate. WGS analysis indicated the presence of *bla*_PER−1_, *bla*_TEM−1D_, *bla*_ADC−31_, and *bla*_OXA−82_. The *bla*_PER−1_ gene was flanked by the putative transposase gene *tpnA1* and *tpnA2* in upstream and downstream region. Insertion sequence IS*Aba1* was located immediate upstream region of AmpC beta-lactamase gene, *bla*_ADC−31_ and carbapenemase gene, *bla*_OXA−82_ (Zander et al., 2013) (Figure S1). Beta-lactam and cephalosporin resistance of this isolate can be clearly argued by the presence of these encoded β-lactamase genes along with the insertion elements, providing the additional promoters for their increased expression (Lin et al., 2010). Resistance to aminoglycosides and gentamicin are contributed by *aac(3*′*)-Ia, aac(6*′*)-Il, aph(3*′*)-Ic*, and *strAB* genes (Tables 1, 2). Levofloxacin resistance was conferred due to the mutations observed in *gyrA* and *parC* genes (Table 3). Twenty to seventy-fold up-regulation of *adeB* and *adeJ* efflux pumps genes were confirmed, which are assumed to contribute to the resistance of levofloxacin, trimethoprim/sulfamethoxazole, tigecycline, clindamycin, chloramphenicol, and tetracyclines (Table 1, Figure 1).
- *Acinetobacter nosocomialis* YMC2003/1/R306 was susceptible to imipenem, meropenem, and ciprofloxacin, intermediate to piperacillin-tazobactam, but resistant to piperacillin, ceftazidime, cefepime, and ampicillin-sulbactam. This isolate is an ideal candidate for ESBL strain, as it carries *bla*_PER−1_,which is identified as a part of composite transposon bracketed by two insertion elements *ISPa12* and *ISPa13*, belonging to IS4 family (Figure S2). Expression of this gene was driven by *ISPa12* promoter, and its genetic environment is similar to the *bla*_PER−1_ found in *Providencia stuartii* and *Pseudomonas aeruginosa* isolates, as reported previously (Yong et al., 2003; Poirel et al., 2005). Efflux pumps showed lower expression, which correlated to its increased susceptibility toward fluoroquinolones and tetracyclines (Tables 1, 3, Figure 1).

**Table 3:** MIC of the fluoroquinolone (ciprofloxacin and levofloxacin) and amino-acid substitutions in the QRDR of the *gyrA, gyrB*, and *parC* genes of panel strains.

	**MIC(μg/ml)**		**Amino-acid substitutions in**		
	CIP^*^	LEV^*^	***gyrA***	***parC***	***gyrB***
**ESBL**					
YMC2003/5/C86	128, R	32, R	S81L	E88K	A677V
YMC2003/1/R306	0.5, S	0.75, R	S81L	–	–
**OVER-EXPRESSED AMPCβ** **-LACTAMASE**					
YMC2009/2/B6756	128, R	6, I	S81L	S81L	–
YMC2012/7/R3167	256, R	8, R	S81L	S81L	–
**OXA-TYPE CARBAPENEMASES**					
YMC2011/7/R812	0.5, S	0.19, S	–	–	–
YMC2012/1/R79	128, R	6, I	S81L	S81L	–
YMC2011/2/C582	128, R	24, R	S81L	S81L	–
YMC2012/9/R2209	256, R	16, R	S81L	S81L	–
**METALLO-β-LACTAMASE**					
YMC2013/3/R2081	128, R	6, I	S81L	S81L	–
**NARROW-SPECTRUM** **β-LACTAMASE**					
YMC2010/8/T346	0.25, S	0.19, S	–	–	–
**NARROW-SPECTRUM OXACILLINASE**					
YMC2009/2/B2968	0.12, S	0.125, S	–	–	–

* *MIC assay was performed using Disk diffusion technique and E-test for ciprofloxacin and levofloxacin, respectively*.

### Over-Expressed AmpC Beta-Lactamase

Overproduction of intrinsic cephalosporinase such as *bla*_ADC−25_, *bla*_ADC−30_, or *bla*_ADC−56_ coupled with insertion elements, such as IS*Aba1*, are responsible for cephalosporin resistance (Lopes and Amyes, 2012).

- *Acinetobacter baumannii* YMC2009/2/B6756 was only susceptible to imipenem and meropenem, but resistant to all other antibiotics and beta-lactamase inhibitor combinations used in the study (Table 1). Genomic analysis indicated the presence of *bla*_TEM−1D_, *bla*_ADC−30_, and *bla*_OXA−66_ (a *bla*_OXA−51−like_ gene) (Figure S3). The *bla*_TEM−1D_ gene in this strain consisted of P3 promoter, which was initially found in Russia contributing to beta-lactam inhibitor-resistance (Edelstein et al., 2000; Leflon-Guibout et al., 2000; Constança and Manuela, 2003). Beta-lactam resistance in this isolate is attributed to the insertion of IS*Aba1* upstream of AmpC gene, *bla*_ADC−30_, mediating its over-expression (Li et al., 2015). OXA-66 is the intrinsic OXA-51 variant class D carbapenemase, which does not confer resistance to carbapenems, although it is associated with IS*Aba1*; however, a point mutation converts it into OXA-82, and this variant confers resistance to imipenem and meropenem (Zander et al., 2013) (Figure S3). OXA-82 and OXA-66 are associated with the International clone 2, which is the most prevalent clone found worldwide (Hu et al., 2007; Evans et al., 2008; Evans and Amyes, 2014). Decreased susceptibility toward levofloxacin, tetracycline, trimethoprim/sulfamethoxazole, rifampicin, chloramphenicol, and gentamicin (Tables 1, 3) is contributed by *aacA4, aadA1, aac(3)-Ia, armA*, and *aac(6')Ib-cr* genes along with the more than 20-fold increased expression of *adeA, adeG, and adeJ* efflux pumps compared to the susceptible strain (Magnet et al., 2001; Coyne et al., 2010; Yoon et al., 2013) (Figure 1).
- *Acinetobacter baumannii* YMC2012/7/R3167 was susceptible to piperacillin-tazobactam, imipenem, and meropenem, but resistant to ampicillin-sulbactam, piperacillin, ceftazidime, cefepime, ceftazidime-clavulanate (Table 1), and ciprofloxacin (Table 3). Whole genome analysis indicated the presence of β-lactamase genes, *bla*_ADC−30_, and *bla*_OXA−66_. (Hu et al., 2007; Zander et al., 2013) (Figure S4). Further analysis indicated the insertion of IS*Aba1* upstream of AmpC gene-*bla*_ADC−30_ which provided a stronger promoter leading to over-expression of AmpC beta-lactamase (Li et al., 2015) leading to multiple beta-lactam resistance. Genetic structure around *bla*_ADC−30_, and *bla*_OXA−66_ of this strain was identical to *A. baumannii* YMC2009/2/B6756. As opposed to the phenotypic resistance scheme for over-expressed AmpC beta-lactamase class, this strain was susceptible to piperacillin-tazobactam, and we were unable to explain the discrepancy for this phenotype. The expressions of *adeB* and *adeG* were similar to *A. baumannii* YMC2009/2/B6756. High-level resistance to tetracycline was observed due to *tet(B)* gene (Takahashi et al., 2002).

### OXA-Type-Carbapenemases

Carbapenem resistance in *Acinetobacter* spp. is mediated by various mechanisms such as membrane impermeability due to loss of porins, but it is mostly mediated by enzymatic hydrolysis of antibiotics (Bou et al., 2000; Quale et al., 2003; Bonomo and Szabo, 2006; Poirel and Nordmann, 2006; Nordmann, 2010). Carbapenem-hydrolyzing class D beta-lactamases (CHDLs) or OXA-type-carbapenemases (OXA-51-like, 23-like, -58-like, -143-like, -40-like, and 235-like), often associated with upstream insertion elements, lead to their over-expression resulting in carbapenem resistance (Poirel et al., 2010). Studies have indicated that OXA-40- and OXA-143-type carbapenemases were not associated with insertion sequences nor integrons (Higgins et al., 2009; Evans and Amyes, 2014). Below we have illustrated the mechanism of few strains expressing OXA-type carbapenemases. According to the resistance determination decision tree, these strains were similar to the phenotype observed in metallo-beta lactamase producers, except its susceptibility toward ceftazidime and cefepime. However, due to the complex resistance mechanism involving multiple beta-lactamases and efflux pumps, most of the strains in this class were resistant to these two antibiotics.

- *Acinetobacter baumannii* YMC2011/7/R812 was susceptible to ceftazidime, ceftazidime-clavulanate, ciprofloxacin, and levofloxacin, but resistant to ampicillin-sulbactam, piperacillin, piperacillin-tazobactam, imipenem, and meropenem (Tables 1, 3). This strain carried CHDLs such as OXA-120, belonging to OXA-51 family, and OXA-23, along with cephalosporinase ADC-77 (Table 2). There were no insertion sequences located around *bla*_OXA−120_ and *bla*_ADC−77_, keeping their expressions at the basal level (Figure S5-A). However, there was an IS*Aba1* insertion upstream of *bla*_OXA−23_ leading to the overexpression of carbapenemase hydrolyzing activity, along with cefepime resistance (Turton et al., 2006; Lin et al., 2010). As illustrated by Naas and Nordmann (2010) and OXA-type carbapenemase detection scheme, these classes of bacteria are susceptible to ceftazidime and cefepime. This strain was susceptible to fluoroquinolones, tetracyclines, and aminoglycosides (Tables 1, 3) due to absence of *adeRS* genes, which encode a two-component system regulating AdeABC expression system. In addition, none of the known aminoglycoside and fluoroquinolone resistant genes were present (Tables 1, 3). In addition, *adeC* gene was also absent, along with truncation of *adeA* gene (Figure S5-B). The genetic structure around *bla*_OXA−120_ from *A. baumannii* YMC2011/7/R812 and *bla*_OXA−66_ from ESBL-producing *A. baumannii* YMC2009/2/B6756 and YMC2012/7/R3167 were identical, as both the beta-lactamases belongs to OXA-51-like group (Rafei et al., 2015).
- *Acinetobacter baumannii* YMC2012/1/R79 was resistant to all of the antibiotics used in this study. This strain carried *bla*_TEM−1D_, *bla*_ADC−30_, *bla*_OXA−66_, and CHDL, *bla*_OXA−23_. The multi-drug resistant phenotype of this strain was contributed by IS*Aba1*-*bla*_OXA−23_ and IS*Aba1-bla*_ADC−30_ genes (Turton et al., 2006; Lin et al., 2010) (Figure S6). Resistance to aminoglycoside were seen due to the presence of *aadA1, aadA24, armA*, and *aac(6*′*)Ib-cr* genes, resistance to fluoroquinolones were due to the mutations in *gyrA* and *parC* genes, along with the moderately increased expressions of *adeB, adeG*, and *adeJ* efflux pumps (Table 1, Figure 1).
- *Acinetobacter baumannii* YMC2011/2/C582 was resistant to all of the antibiotics and beta-lactam inhibitors used in this study for phenotypic screening (Table 1). WGS analysis indicated the presence of ESBL gene, *bla*_PER−1_, and wide variety of other β-lactamase genes, such as *bla*_OXA−66_, *bla*_OXA−23_, and *bla*_ADC−30_ (Table 2). The *bla*_PER−1_ gene and partial *glutathione-S-transferase* were bracketed by IS*Pa12* and IS*Pa13*, belonging to the IS4 family, regulating the expression of *bla*_PER−1_ gene driven by promoter sequences in IS*Pa12* (Poirel et al., 2005), similar to *A. nosocomialis* YMC2003/1/R306 strain (Figures S2, S7). In addition, there was insertion of IS*Aba1* upstream of *bla*_ADC−30_ and *bla*_OXA−23_, providing additional promoter leading to increased resistance due to overexpressions of AmpC beta-lactamase and carbapenemase, respectively. Increased expression of *adeb, adeG*, and *adeJ*, along with aminoglycoside and fluoroquinolones resistance genes such as *armA, aph(3*′*)-Ic, strAB, aph(3*′*)-VIb, aadA1*, and *aac(6*′*)Ib-cr* decreased the susceptibility toward gentamicin, tetracycline, trimethoprim/sulfamethoxazole, rifampicin, and chloramphenicol (Table 1, Figure 1). In addition, mutations were observed in *gyrA* and *parC* genes, which caused levofloxacin resistance (Table 3).
- *Acinetobacter baumannii* YMC2012/9/R2209 was intermediate to imipenem but resistant to all other cephalosporin and carbapenems used in our study (Table 1). This isolate was AmpC beta-lactamase hyper-producer along with CHDL, which was revealed by the presence of IS*Aba1-bla*_OXA−82_ and IS*Aba1*-*bla*_ADC−30_ (Figure S8). Increased carbapenem resistance was caused by IS*Aba1-bla*_OXA−82_ (Zander et al., 2013). Susceptibility toward tigecycline, gentamicin and tetracycline were due to the absence of aminoglycoside resistance genes and lower expressions of *adeB* and *adeG* efflux pumps (Table 1, Figure 1). In contrast, increased relative expression of *adeJ* gene might have increased resistance to fluoroquinolones, such as ciprofloxacin and levofloxacin, along with *gyrA* and *parC* genes mutations.

### MBL

MBL-producing *Acinetobacter* spp. have become an emerging therapeutic concern worldwide. Along with CHDLs, carbapenem resistance is attributed to MBLs such as IMP, VIM, GIM, SIM etc. (Kim et al., 2014). According to the resistance detection scheme, *Acinetobacter* spp. producing MBLs display similar phenotypic resistance as OXA-type carbapenemases, except the latter showing its susceptibility toward ceftazidime and cefepime. MBL producing *A. pittii* YMC2013/3/R2081 was susceptible to piperacillin-tazobactam and imipenem but resistant to ampicillin-sulbactam, piperacillin, ceftazidime, cefepime, meropenem, ceftazidime-clavulanate, and ciprofloxacin. This bacterium contains *bla*_CARB−8_, *bla*_PER−1_, *bla*_ADC−18_, *bla*_OXA−500_, and *bla*_SIM−1_ (Tables 1, 2). Resistance to most of antibiotics can be explained due to ESBL gene along with IS element, *IS*CR1-*bla*_PER−1_, and MBL gene, *bla*_SIM−1_ (Figure S9). Despite SIM-1 production, this bacterium was susceptible to imipenem due to its strong activity against *Acinetobacter* spp (Lee et al., 2005). Genetic analysis indicated that *bla*_SIM−1_ along with *aar-3, carB3*, and *aadA1* genes were encoded by class 1 integron. The *bla*_CARB−8_ is carbencillin-hydrolyzing beta-lactamase, which has the same hydrolytic profile as *bla*_CARB−5_ (Choury et al., 2000). This enzyme has been previously identified in various species such as *Oligella urethralis, Vibrio cholerae, Achromobacter xylosoxidans, A. baumannii*, and *Salmonella typhimurium*, which indicates inter-genus transferability of the gene (Decre et al., 1995; Ridley and Threlfall, 1998; Choury et al., 1999, 2000; Lin et al., 2010). Increased resistance to gentamicin was mediated by the *aac(3)-IId* gene (Ho et al., 2010), despite lower expression of *adeB, adeG*, and *adeJ* efflux pumps (Table 1, Figure 1).

### Narrow Spectrum β-Lactamase

*Acinetobacter pittii* YMC2010/8/T346 belongs to a novel sequence type 1385 (ST 1385), and is susceptible to ampicillin-sulbactam, piperacillin-tazobactam, ceftazidime, cefepime, imipenem, ceftazidime-clavulanate, and ciprofloxacin but resistant to meropenem (Tables 1, 3). Sequence analysis indicated the presence of *bla*_OXA−506_, variant of *A. pittii* intrinsic *bla*_OXA−213−like_*, bla*_ADC−41_, and *bla*_OXA−499_, which were not associated with insertion elements (Table 2, Figure S10). The *bla*_OXA−499_ is a novel variant of carbapenem hydrolyzing oxacillinase, *bla*_OXA−143_. This gene was first found in South Korea, and is the carbapenem hydrolyzing gene which explains its resistance to the meropenem, as reported previously (D'Souza et al., 2017). Wide susceptibility toward aminoglycosides, tetracyclines, and fluoroquinolones was observed due to the lower expression of efflux pumps and absence of any corresponding resistance genes (Tables 1, 3, Figure 1).

### Narrow Spectrum Oxacillinase

*Acinetobacter pittii* YMC2009/2/B2968 belonging to novel ST1638, was not resistant to the antibiotics tested in this study (Table 1). Whole genome analysis revealed *bla*_OXA−421_, a CHDL belonging to *A. pittii* intrinsic *bla*_OXA−213_ family and *bla*_ADC−22_ (Table 2, Figure S11). However, no existing study has yet demonstrated that the carbapenemase activity of *bla*_OXA421._ *bla*_ADC−22_ is a naturally occurring cephalosporinase gene in *A. baumannii*, which is repressed under normal conditions (Beceiro et al., 2009; Li et al., 2015). This strain exhibited the highest susceptibility toward aminoglycosides, tetracyclines, and fluoroquinolones among all other panel strains, due to the absence of corresponding resistance genes and lowest expression of efflux pumps. Therefore, this was selected as the reference strain to calculate the relative expression of efflux pumps for other strains.

### Analysis of QRDRs for *gyrA* and *parC* Genes and Fluoroquinolone Resistance

The MICs of ciprofloxacin and levofloxacin were determined (Table 3). Both antibiotics functioned by inhibiting DNA gyrase subunit A (GyrA), DNA gyrase subunit B (GyrB), and toposiomerase IV subunit C (ParC) (Drlica and Zhao, 1997), and hence exhibited similar resistance phenotypes for the panel strains. Resistance to fluoroquinolone in bacteria was mediated by spontaneous mutations in *gyrA, gyrB*, and *parC* genes (Park et al., 2011; Ardebili et al., 2015). We identified the substitutions in GyrA (Ser81Leu) and ParC (Ser84Leu) in all fluoroquinolone resistant strains (Table 3). Ser467Gly and Glu88Lys mutation in ParC did not correlate with the resistance phenotypes. As opposed to the previous studies, we found GyrA (Ser81Leu) and ParC (Ser467Gly) mutations in *A. nosocomialis* YMC2003/1/R306, which were susceptible to fluoroquinolone (Vila et al., 1995). We could not find Glu479Asp, Cys423Ser, Glu479Asp, Leu420Gln, Cys423Ser, Leu433His, Glu479Asp, and D644Y mutations in GyrB which were previously described as novel substitutions (Park et al., 2011), except A677V in *A. baumannii* YMC2003/5/C86.

### Efflux-Mediated Antimicrobial Resistance

Overexpression of efflux pumps are one of the major mechanisms that contribute to the multidrug resistance in *Acinetobacter* species. Genes encoding these systems are carried by mobile genetic elements or chromosomes, and thus be responsible for acquired or intrinsic resistance (Coyne et al., 2011). Five categories of efflux pump systems have been described, which are responsible for pumping out diverse classes of antibiotics: resistance-nodulation-cell division (RND) family, ATP-binding cassette (ABC) transporters, major facilitator superfamily (MFS), small multidrug resistance (SMR) family, and the recently identified multidrug and toxic compound extrusion (MATE) family (Piddock, 2006; Vila et al., 2007). Considering the broad-range substrate specificity of the three RND-type efflux pump systems, AdeABC, AdeFGH, and AdeIJK, we investigated the expressions of *adeB, adeG*, and *adeJ* genes (Figures 1A–C). Reference gene *rpoB* was used as a control, and susceptible strain *A. pittii* YMC2009/2/B2968 was used as a reference. Tigecycline appeared to be the best substrate for *adeB* pump, which correlated with their increased resistance and seven to 50-fold increase in its expression. This was consistent with previous findings (Perez et al., 2007; Ruzin et al., 2007; Hornsey et al., 2010) (Table 1, Figure 1). In addition, decreased susceptibility toward tetracycline, trimethoprim/sulfamethoxazole, and gentamicin also correlated with the increased expression with few exceptions. We screened for mutations in AdeRS, a two-component regulator system that controls the expression of AdeRS. G186V substitution in AdeS and A136V in AdeR was detected in all of the isolates overexpressing *adeB* gene, which was previously linked to increased tigecycline resistance (Hornsey et al., 2010; Rumbo et al., 2013) (Tables S3, S4). The isolate *A. baumannii* YMC2011/7/R812 did not contain *adeRS, adeA*, and *adeC* genes (Table S2). The *adeC* gene was also absent from *A. baumannii* YMC2012/9/R2209 and all *A. pittii* strains (Table S2). The expressions of *adeG* and *adeJ* were variable and strain-specific. Therefore, we could not find the suitable phenotypic marker regulating the pump. Overall, *A. baumannii* isolates showed increased expression of three RND efflux systems compared to *A. pittii* and *A. nosocomialis*. AdeFGH and its LysR-type transcriptional regulator AdeL were present in all strains (Table S5). TetR transcriptional repressor AdeN, controlling AdeIJK were interrupted by IS*Aba1* insertion sequence in *A. baumannii* YMC2012/9/R2209, YMC2012/7/R3167, and YMC2011/2/C582, which increased AdeIJK expression (Rosenfeld et al., 2012) (Table S5). In addition, we were unable to correlate the expression of BaeSR two-component system, which was previously known to influence tigecycline susceptibility by regulating *adeABC* genes (Lin et al., 2014) (Figure 1D). The limitation of our qRT-PCR was using different primers for different species due to the polymorphism identified in efflux pumps. This might have led to different amplicon kinetics resulting in errors in differential expressions. Finally, we could also detect the genes related to non-RND efflux pumps such as *cra, amvA, abeM, abeS*, and *adeXYZ* in all of the *Acinetobacter* strains. The *adeDE* gene was identified in YMC2003/1/R306 and YMC2013/3/R2081, and c*mlA* was present only in isolate YMC2013/3/R2081 (Table S6).

### Role of Porins in Resistance

Porins play a vital role in the mechanism of carbapenem resistance in *Enterobacteriaceae*. However, in *Acinetobacter* spp., their contributions toward resistance are debated, and their functions remain ambiguous (Marti et al., 2006). Previous studies indicated that loss of porins such as *CarO, OprD*, and *33-36Kda Omp* conferred carbapenem resistance (Bou et al., 2000; Fernandez-Cuenca et al., 2003; Mussi et al., 2005; Siroy et al., 2005; Peleg et al., 2008). To determine the potential role of these porins in resistance, we performed SDS-PAGE (data not shown) and MALDI-TOF (Figure S12). All of the panel strains showed identical OMP profiles, which were also confirmed by WGS analysis (Table S7). These results suggested that the porins did not have any role in carbapenem resistance among the panel strains. In addition, qRT-PCR for *CarO, oprD*, and *33-36Kda Omp* did not show any significant correlation to antimicrobial resistance (Figure S13).

### Virulence Factors

Understanding the pathogenesis, along with its multi-drug resistance phenotype, is highly essential for infection control and investigation of alternate treatment options. The development of infection, and bacterial survival in the host depends on virulence factors such as biofilm formation, serum resistance, evasion of the host immune response, motility, host cell apoptosis, bacterial dissemination, transfer of genetic material between bacterial cells, and iron acquisition mechanisms (Choi et al., 2005; Jacobs et al., 2010; Luke et al., 2010; Jin et al., 2011; Gaddy et al., 2012; McConnell et al., 2013). Virulence factors capsular polysaccharide (*ptk* and *epsA*), phospholipase D, and penicillin-binding protein (*pbpG*) were present in all of the panel strains (Table S8). Virulent genes associated with biofilm formation, such as *OmpA* and BfmR, the response regulator component of two-component system BfmRS, were present in all of the strains (Gaddy et al., 2009; Liou et al., 2014). However, another key virulent gene, *bap* (Biofilm-associated protein), was absent in YMC2011/7/R812, YMC2012/9/R2209, YMC2013/3/R2081, and YMC2009/2/B2968 (Badmasti et al., 2015). Outer membrane proteins, CsuA/B, CsuC, and CsuD were absent from YMC2011/7/R812 and YMC2010/8/T346. *Acinetobacter nosocomialis* YMC2003/1/R306 did not carry the genes involved in acinetobactin-mediated iron acquisition system such as *bauA, bauB, bauC, bauD, bauE, basC*, and *basD*, and we did not find homologs of these systems either.

In summary, all of the panel strains in our study were shortlisted depending on the resistance scheme given by François et al. (2004) and Naas and Nordmann in Antibiogram (Naas and Nordmann, 2010). Similar to our previous study in *Klebsiella pneumoniae* (Dsouza et al., 2017), we found several discrepancies in the detection scheme. The ESBL strain YMC2003/5/C86 isolated in our study was resistant to carbapenems due to presence of OXA-82, albeit the scheme indicates that ESBL strains should be susceptible to carbapenems. Similarly, it also indicates that OXA-type carbapenemases are susceptible to ceftazidime and cefepime. However, the isolated strains in this study were resistant to both antibiotics. Therefore, we suggest the scheme to be updated and modified considering the novel mutations, acquisition of multiple resistance genes, and transposon insertion, for better detection. The main drawback of this study was characterizing unequal number of strains in each resistance classes. Strains were obtained retrospectively and therefore, limiting the number of strains.

The basic rule in the pharmaceutical industry for developing new antibiotics, or for clinicians prescribing antibacterial therapy, depends on comprehensive understanding of the mechanism(s) of resistance. For some time now, *Acinetobacter* spp. have been implicated in several pathological conditions, and constant efforts are being undertaken to control the spread of these organisms in hospital and community settings (Maragakis and Perl, 2008; Vila and Pachon, 2008; Metan et al., 2009; Garnacho-Montero and Amaya-Villar, 2010; Evans et al., 2013; Wisplinghoff and Seifert, 2014; Dramowski et al., 2015). There are several mechanisms suggested for *Acinetobacter* spp. resistance for β-lactams and other antibiotics that we have outlined in this study. In hospital settings and research laboratories, it is quite common to encounter these pathogens with various resistance phenotypes. The genotypic and phenotypic correlations in our study would definitely help clinicians and researchers to better understand the mechanism associated, along with utilizing these pathogens as reference strains. In addition, these panel strains would be highly beneficial for evaluating the efficacy of novel antibiotics or antibiotic kit on *Acinetobacter* spp. displaying different resistance phenotypes. An in-depth study involving the genetic mechanism conferring resistance can open many opportunities for novel drug target study and ways to control the antimicrobial resistance. We have studied the role of various resistance genes attributing to the specific resistance in detail, by referring to previous publications. Therefore, we believe that we have constructed a single platform consisting of various resistance genes illustrating its role, which can help antimicrobial researchers to understand the basics of antimicrobial resistance. Further, studies could be warranted to determine the lineage analysis on this strain and also understand the expression of virulence factors contributing toward the bacterial pathogenesis.

## Author Contributions

DY, JC, and YC designed the study and secured the funding. RD, NP, NLP, and TV performed the experiments. RD, PH, J-HB, and DY analyzed and interpreted the data and wrote the manuscript.

### Conflict of Interest Statement

The authors declare that the research was conducted in the absence of any commercial or financial relationships that could be construed as a potential conflict of interest.
