# Supplementary material for: Phenotypic and Genotypic Characterization of Acinetobacter spp. Panel Strains: A Cornerstone to Facilitate Antimicrobial Development
Source: Front Microbiol. 2019 Mar 26;10:559. doi: 10.3389/fmicb.2019.00559 (PMC6446969; doi:10.3389/fmicb.2019.00559)
Supplement: Supplementary file 1 [file Data_Sheet_1.docx]

| Panel strains in each phenotypes | ST | PIP | PIP-TZ | CAZ | FEP | IPM | MEM | CAZ-CLV | SAM |
| --- | --- | --- | --- | --- | --- | --- | --- | --- | --- |
| ESBL |  | **R** | **I/R** | **R** | **R** | **S** | **S** |  |  |
| *A. baumannii*YMC2003/5/C86 | 423 | 256,R | 128,R | 64,R | 64,R | 32,R | 32,R | 8,S | 32,R |
| *A. nosocomialis* YMC2003/1/R306 | 948 | 256,R | 32,I | 64,R | 64,R | 2,S | 4,S | 4,S | 32,R |
| Over-expressed AmpC β-lactamase |  | **I/R** | **I/R** | **R** | **I/R** | **S** | **S** |  |  |
| *A. baumannii*YMC2009/2/B6756 | 191 | 256,R | 256,R | 256,R | 32,R | 2,S | 4,S | 64,R | 32,R |
| *A. baumannii*YMC2012/7/R3167 | 208 | 256,R | 8,S | 64,R | 32,R | 1,S | 4,S | 64,R | 32,R |
| *A. baumannii*YMC2009/3/B7798 |  | 256,R | 1,S | 256,R | 128,R | 2,S | 2,S | 64,R | 32,R |
| *A.Baumannii*YMC2009/6/P319 |  | 256,R | 1,S6 | 256,R | 128,R | 4,S | 64,R | 64,R | 128,R |
| *A.Baumannii*YMC2009/3/R1487 |  | 32,I | 256,R | 128,R | 64,R | 32,R | 32,R | 128,R | 128,R |
| *A.Baumannii*YMC2009/7/B8464 |  | 256,R | 1,S | 256,R | 32,R | 2,S | 2,S | 64,R | 32,R |
| Oxa-carbapenemase |  | **R** | **R** | **S** | **S** | **I/R** | **I/R** |  |  |
| *A.Baumannii*YMC2011/2/C582 | 208 | 256,R | 256,R | 256,R | 128,R | 256,R | 128,R | 64,R | 128,R |
| *A.Baumannii*YMC2011/7/R812 | 1386 | 256,R | 256,R | 4,S | 16,I | 16,R | 32,R | 4,S | 32,R |
| *A.Baumannii*YMC2012/1/R79 | 191 | 256,R | 256,R | 256,R | 128,R | 32,R | 64,R | 64,R | 32,R |
| *A.Baumannii*YMC2012/9/R2209 | 229 | 256,R | 256,R | 256,R | 64,R | 8,I | 32,R | 64,R | 32,R |
| *A.Baumannii*YMC2011/7/U2158 |  | 256,R | 256,R | 4,S | 16,I | 16,R | 32,R | 4,S | 32,R |
| *A.Baumannii*YMC2011/7/P479 |  | 256,R | 256,R | 16,I | 8,S | 8,I | 64,R | 8,S | 8,S |
| *A.Baumannii*YMC2010/9/R966 |  | 256,R | 256,R | 16,I | 16,I | 32,R | 32,R | 4,S | 8,S |
| *A.pittii*YMC2012/1/U133 |  | 256,R | 256,R | 64,R | 128,R | 32,R | 32,R | 64,R | 128,R |
| *A.Baumannii*YMC2010/10/R659 |  | 256,R | 256,R | 16,I | 16,I | 32,R | 64,R | 16,I | 16,R |
| *A.Baumannii*YMC2012/1/U17 |  | 256,R | 0.5,S | 256,R | 128,R | 64,R | 64,R | 64,R | 64,R |
| Metallo-β-lactamase |  | **R** | **R** | **R** | **R** | **R** | **R** |  |  |
| *A.pittii*YMC2013/3/R2081 | 1030 | 256,R | 4,S | 256,R | 64,R | 4,S | 64,R | 32,R | 16,R |
| *A.Baumannii*YMC2013/2/U2800 |  | 256,R | 64,I | 32,R | 32,R | 32,R | 64,R | 32,R | 16,R |
| *A.Baumannii*YMC2012/3/U2025 |  | 128,R | 64,I | 32,R | 8,S | 32,R | 128,R | 32,R | 128,R |
| *A.Baumannii*YMC2011/6/U1483 |  | 256,R | 0.5,S | 8,S | 128,R | 32,R | 64,R | 32,R | 64,R |
| *A.Baumannii*YMC2012/9/U3624 |  | 256,R | 0.5,S | 8,S | 128,R | 128,R | 256,R | 32,R | 128,R |
| Narrow-spectrum β-lactamase |  | **I/R** | **I/R** | **S** | **S** | **S** | **S** |  |  |
| *A.pittii* YMC2010/8/T346 | 1385 | 32,I | 0.5,S | 4,S | 4,S | 2,S | 16,R | 4,S | 4,S |
| *A.Baumannii*YMC2011/6/R1565 |  | 32,I | 0.5,S | 256,R | 4,S | 0.25,S | 2,S | 4,S | 4,S |
| Narrow-spectrum β-lactamase |  | **R** | **R** | **S** | **S** | **S** | **S** |  |  |
| *A.pittii*YMC2009/2/B2968 | 1638 | 32,I | 0.5,S | 4,S | 4,S | 0.25,S | 0.5,S | 4,S | 2,S |
| Wild type |  | **S** | **S** | **S** | **S** | **S** | **S** |  |  |
| YMC2013/1/R3000 |  | 256,R | 0.5,S | 8,S | 16,I | 0.25,S | 1,S | 4,S | 4,S |

Table S1: Complete list of *Acinetobacter* spp. and its MIC (**(**μg/mL, interpretation).

Note: MLST, Multilocus sequence typing; R, Resistant; I, Intermediate; S, susceptible; PIP, piperacillin; PIP/TZ, piperacillin-tazobactam; CAZ, ceftazidime; FEP, cefepime; IPM, imipenem; MER, meropenem; CIP, ciprofloxacin, CAZ/CLV, ceftazidime-clavulanate; SAM, Ampicillin/Sulbactam.

Table S2: Profile of AdeABC efflux pump along with its transcriptional regulators AdeRS in *Acinetobacter* isolates.

| Panel strains in each phenotypes | *adeR* | *adeS* | *adeA* | *adeB* | *adeC* |
| --- | --- | --- | --- | --- | --- |
| ESBL |  |  |  |  |  |
| YMC2003/5/C86 | + | + | + | + | + |
| YMC2003/1/R306 | + | + | + | + | + |
| Over-expressed AmpC β-lactamase |  |  |  |  |  |
| YMC2009/2/B6756 | + | + | + | + | + |
| YMC2012/7/R3167 | + | + | + | + | + |
| OXA-type carbapenemase |  |  |  |  |  |
| YMC2011/7/R812 | - | - | - | + | - |
| YMC2012/1/R79 | + | + | + | + | + |
| YMC2011/2/C582 | + | + | + | + | + |
| YMC2012/9/R2209 | + | + | + | + | - |
| Metallo-β-lactamase |  |  |  |  |  |
| YMC2013/3/R2081 | + | + | + | + | - |
| Narrow-spectrum β-lactamase |  |  |  |  |  |
| YMC2010/8/T346 | + | + | + | + | - |
| Narrow-spectrum oxacillinase |  |  |  |  |  |
| YMC2009/2/B2968 | + | + | + | + | - |

| Panel strains | TM | |  | S |  | HAMP | | |  | Dhp | | | | | | | | | |  | CA | | | | | | | | | | | | | | |
| --- | --- | --- | --- | --- | --- | --- | --- | --- | --- | --- | --- | --- | --- | --- | --- | --- | --- | --- | --- | --- | --- | --- | --- | --- | --- | --- | --- | --- | --- | --- | --- | --- | --- | --- | --- |
|  | **27V** | **32V** |  | **48F** |  | **94A** | **125N** | **139N** |  | **143W** | **144N** | **149H** | **152R** | **163Q** | **186G** | **189H** | **214F** | **227D** | **235V** |  | **252I** | **N** | **268N** | **280S** | **281Q** | **G1** | **299Q** | **F** | **G2** | **331I** | **336G** | **337T** | **348V** | **352K** | **354S** |
| C86 | V | V |  | F |  | A | N | N |  | W | N | H | R | Q | **V** | H | F | D | V |  | I |  | H | S | Q |  | Q |  |  | I | G | T | I | K | S |
| R306 | L | L |  | I |  | A | N | N |  | W | N | H | R | Q | G | H | F | D | V |  | I |  | N | S | Q |  | R |  |  | I | G | T | I | K | S |
| B6756 | V | V |  | F |  | A | N | N |  | W | N | H | R | Q | V | H | F | D | V |  | I |  | H | S | Q |  | Q |  |  | I | G | T | I | K | S |
| R3167 | V | V |  | F |  | A | N | N |  | W | N | H | R | Q | V | H | F | D | V |  | I |  | H | S | Q |  | Q |  |  | I | G | T | I | K | S |
| R812 | - | - |  | - |  | - | - | - |  | - | - | - | - | - | - | - | - | - | - |  | - | - | - | - | - | - | - | - | - | - | - | - | - | - | - |
| R79 | V | V |  | F |  | R | N | N |  | W | N | H | R | Q | V | H | F | D | V |  | I |  | H | S | Q |  | Q |  |  | I | G | T | I | K | S |
| C582 | V | V |  | F |  | A | N | N |  | W | N | H | R | Q | V | H | F | D | V |  | I |  | H | S | Q |  | Q |  |  | I | G | T | I | K | S |
| R2209 | V | V |  | F |  | A | N | N |  | W | N | H | R | Q | G | Y | F | D | I |  | I |  | N | S | Q |  | Q |  |  | I | G | T | V | K | P |
| R2081 | L | G |  | L |  | A | N | N |  | W | N | H | R | Q | G | N | F | E | K |  | I |  | N | Q | T |  | R |  |  | V | G | Q | I | S | P |
| T346 | L | G |  | L |  | A | N | N |  | W | N | H | R | Q | G | N | F | E | K |  | I |  | N | Q | T |  | R |  |  | V | G | Q | I | S | P |
| B2968 | L | G |  | L |  | A | N | N |  | W | N | H | R | Q | G | N | F | E | K |  | I |  | N | Q | T |  | R |  |  | V | G | Q | I | S | P |

Table S3: Amino acid substitutions in AdeS.

Table S4: Amino acid substitutions in AdeR.

| **Panel strains** | **REC** | | | | | | | | | | **Output** | | | | | | | |
| --- | --- | --- | --- | --- | --- | --- | --- | --- | --- | --- | --- | --- | --- | --- | --- | --- | --- | --- |
|  | **14V** | **D** | **56P** | **63D** | **D** | **K** | **120V** | **134N** | **136A** | **137T** | **142L** | **158H** | **175I** | **192L** | **195H** | **219E** | **228I** | **243V** |
| **C86** | V |  | P | D |  |  | I | N | V | T | L | H | I | L | H | S | I | V |
| **R306** | V |  | P | D |  |  | V | N | A | S | I | H | I | L | H | S | I | V |
| **B6756** | V |  | P | D |  |  | I | N | V | T | L | H | I | L | H | S | I | V |
| **R3167** | V |  | P | D |  |  | I | N | V | T | L | H | I | L | H | S | I | V |
| **R812** | - | - | - | - | - | - | - | - | - | - | - | - | - | - | - | - | - | - |
| **R79** | V |  | P | D |  |  | I | N | V | T | L | H | I | L | H | S | I | V |
| **C582** | V |  | P | D |  |  | I | N | V | T | L | H | I | L | H | S | I | V |
| **R2209** | V |  | P | D |  |  | V | N | A | T | L | H | I | L | H | S | I | V |
| **R2081** | L |  | P | D |  |  | V | Q | T | P | I | H | I | L | Q | S | I | K |
| **T346** | L |  | P | D |  |  | V | Q | T | P | I | H | I | L | Q | S | I | K |
| **B2968** | L |  | P | D |  |  | V | Q | T | P | I | H | I | L | Q | S | I | K |

Table S5: Profile of adeN-adeIJK, adeL-adeFGH and BaeSR in *Acinetobacter* isolates.

|  | **adeN** | **adeIJK** | **adeL** | **adeFGH** | **baeSR** |
| --- | --- | --- | --- | --- | --- |
| **ESBL** |  |  |  |  |  |
| YMC2003/5/C86 | + | + | + | + | + |
| YMC2003/1/R306 | + | + | + | + | + |
| **Over-expressed AmpC β-lactamase** |  |  |  |  |  |
| YMC2009/2/B6756 | + | + | + | + | + |
| YMC2012/7/R3167 | ISAba1 | + | + | + | + |
| **Oxa-type carbapenemase** |  |  |  |  |  |
| YMC2011/7/R812 | + | + | + | + | + |
| YMC2012/1/R79 | + | + | + | + | + |
| YMC2011/2/C582 | ISAba1 | + | + | + | + |
| YMC2012/9/R2209 | ISAba1 | + | + | + | + |
| **Metallo-β-lactamase** |  |  |  |  |  |
| YMC2013/3/R2081 | + | + | + | + | + |
| **Narrow-spectrum β-lactamase** |  |  |  |  |  |
| YMC2010/8/T346 | + | + | + | + | + |
| **Narrow-spectrum oxacillinase** |  |  |  |  |  |
| YMC2009/2/B2968 | + | + | + | + | + |

Table S6: Non-RND efflux-pump systems in *Acinetobacter* spp.

|  | **CrA** | **AmvA** | **AbeM** | **AbeS** | **AdeXYZ** | **AdeDE** | **CmlA** | **FLoR** |
| --- | --- | --- | --- | --- | --- | --- | --- | --- |
| **ESBL** |  |  |  |  |  |  |  |  |
| YMC2003/5/C86 | + | + | + | + | + | - | - | - |
| YMC2003/1/R306 | + | + | + | + | + | + | - | - |
| **Over-expressed AmpC β-lactamase** |  |  |  |  |  |  |  |  |
| YMC2009/2/B6756 | + | + | + | + | + | - | - | - |
| YMC2012/7/R3167 | + | + | + | + | + | - | - | - |
| **OXA-type carbapenemase** |  |  |  |  |  |  |  |  |
| YMC2011/7/R812 | + | + | + | + | + | - | - | - |
| YMC2012/1/R79 | + | + | + | + | + | - | - | - |
| YMC2011/2/C582 | + | + | + | + | + | - | - | - |
| YMC2012/9/R2209 | + | + | + | + | + | - | - | - |
| **Metallo-β-lactamase** |  |  |  |  |  |  |  |  |
| YMC2013/3/R2081 | + | + | + | + | + | + | + | - |
| **Narrow-spectrum β-lactamase** |  |  |  |  |  |  |  |  |
| YMC2010/8/T346 | + | + | + | + | + | - | - | - |
| **Narrow-spectrum oxacillinase** |  |  |  |  |  |  |  |  |
| YMC2009/2/B2968 | + | + | + | + | + | - | - | - |

Table S7: Outer membrane proteins.

|  | **CarO** | **33-36Kda** | **OprD** |
| --- | --- | --- | --- |
| **ESBL** |  |  |  |
| YMC2003/5/C86 | + | + | + |
| YMC2003/1/R306 | + | + | + |
| **Over-expressed AmpC β-lactamase** |  |  |  |
| YMC2009/2/B6756 | + | + | + |
| YMC2012/7/R3167 | + | + | + |
| **Oxa-type carbapenemase** |  |  |  |
| YMC2011/7/R812 | + | + | + |
| YMC2012/1/R79 | + | + | + |
| YMC2011/2/C582 | + | + | + |
| YMC2012/9/R2209 | + | + | + |
| **Metallo-β-lactamase** |  |  |  |
| YMC2013/3/R2081 | + | + | + |
| **Narrow spectrum β-lactamase** |  |  |  |
| YMC2010/8/T346 | + | + | + |
| **Narrow-spectrum oxacillinase** |  |  |  |
| YMC2009/2/B2968 | + | + | + |

Table S8: Virulence and pathogenesis factors in *Acinetobacter* strains.

| Virulence factor | Biofilm  formation | | | Capsular polysaccharide | | Phospholipase D | Penicillin-binding protein 7/8 | Outer membrane vesicles | | | | | | | | | | | Acinetobactin-mediated iron acquisition system | | | | | | |
| --- | --- | --- | --- | --- | --- | --- | --- | --- | --- | --- | --- | --- | --- | --- | --- | --- | --- | --- | --- | --- | --- | --- | --- | --- | --- |
| Gene | ompA | Bfmr-bfms | bap | ptk | epsA | Phospholipase D | pbpG | Csu  A/B | CsuC | CsuD | Putative hemolysin | Putative serine protease | Cu/Zn superoxide dismutase | Fimbrial protein | Bacterioferritin | RND superfamily-like exporter | Putative RND type efflux pump | Putative protease | bauD | bauC | bauE | bauB | bauA | basC | basD |
| C86 | **+** | **+** | **+** | **+** | **+** | **+** | **+** | **+** | **+** | **+** | **+** | **+** | **+** | **+** | **+** | **+** | **+** | **+** | **+** | **+** | **+** | **+** | **+** | **+** | **+** |
| R306 | **+** | **+** | **+** | **+** | **+** | **+** | **+** | **+** | **+** | **+** | **+** | **+** | **+** | **+** | **+** | **-** | **+** | **+** | **-** | **-** | **-** | **-** | **-** | **-** | **-** |
| B6756 | **+** | **+** | **+** | **+** | **+** | **+** | **+** | **+** | **+** | **+** | **+** | **+** | **+** | **+** | **+** | **+** | **+** | **+** | **+** | **+** | **+** | **+** | **+** | **+** | **+** |
| R3167 | **+** | **+** | **+** | **+** | **+** | **+** | **+** | **+** | **+** | **+** | **+** | **+** | **+** | **+** | **+** | **+** | **+** | **+** | **+** | **+** | **+** | **+** | **+** | **+** | **+** |
| R812 | **+** | **+** | **-** | **+** | **+** | **+** | **+** | **+** | **+** | **+** | **+** | **+** | **+** | **+** | **+** | **-**(inser) | **+** | **+** | **+** | **+** | **+** | **+** | **+** | **+** | **+** |
| R79 | **+** | **+** | **+** | **+** | **+** | **+** | **+** | **+** | **+** | **+** | **+** | **+** | **+** | **+** | **+** | **+** | **+** | **+** | **+** | **+** | **+** | **+** | **+** | **+** | **+** |
| C582 | **+** | **+** | **+** | **+** | **+** | **+** | **+** | **-** | **-** | **-** | **+** | **+** | **+** | **+** | **+** | **+** | **+** | **+** | **+** | **+** | **+** | **+** | **+** | **+** | **+** |
| R2209 | **+** | **+** | **-** | **+** | **+** | **+** | **+** | **+** | **+** | **+** | **+** | **+** | **+** | **+** | **+** | **+** | **+** | **+** | **+** | **+** | **+** | **+** | **+** | **+** | **+** |
| R2081 | **+** | **+** | **-** | **+** | **+** | **+** | **+** | **+** | **+** | **+** | **+** | **+** | **+** | **+** | **+** | **+** | **+** | **+** | **+** | **+** | **+** | **+** | **+** | **+** | **+** |
| T346 | **+** | **+** | **+** | **+** | **+** | **+** | **+** | **-** | **-** | **-** | **+** | **+** | **+** | **+** | **+** | **+** | **+** | **+** | **+** | **+** | **+** | **+** | **+** | **+** | **+** |
| B2968 | **+** | **+** | **-** | **+** | **+** | **+** | **+** | **+** | **+** | **+** | **-** | **+** | **+** | **+** | **+** | **-**(inser) | **+** | **+** | **+** | **+** | **+** | **+** | **+** | **+** | **+** |


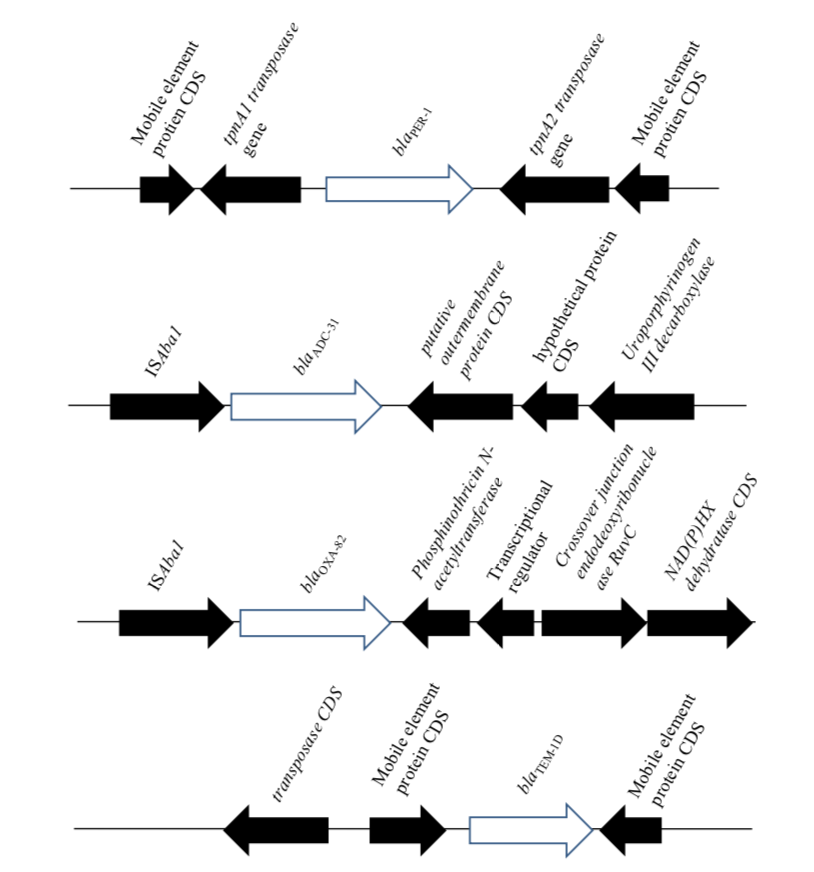


Figure S1: *A. baumannii* YMC2003/5/C86 expressing *bla*_PER-1_, *bla*_ADC-31_ *bla*_OXA-82_, and *bla*_TEM-1D_.


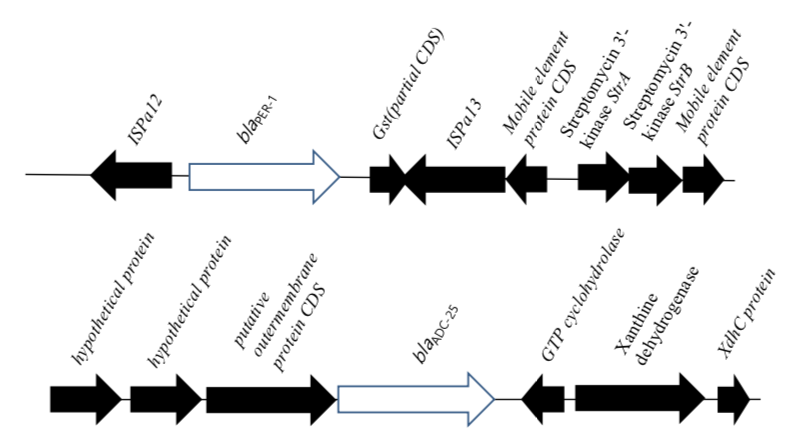


Figure S2: *A. nosocomialis* YMC2003/1/R306 expressing *bla*_PER-1_ and *bla*_ADC-25._


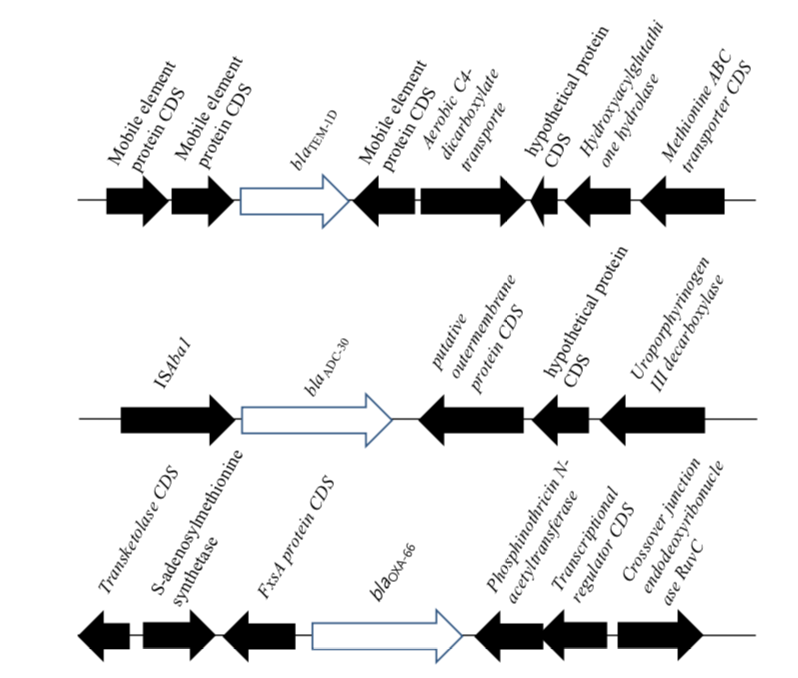


Figure S3: *A. baumannii* YMC2009/2/B6756 expressing *bla*_TEM-1D_, *bla*_ADC-30_ and *bla*_OXA-66_ (a bla_OXA-51-like_ gene_)_


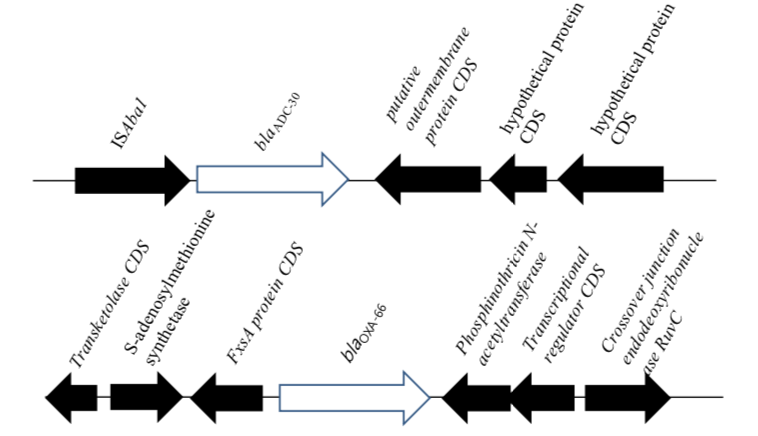


Figure S4: *A. baumannii* YMC2012/7/R3167 expressing *bla*_OXA-66_ and *bla*_ADC-30._


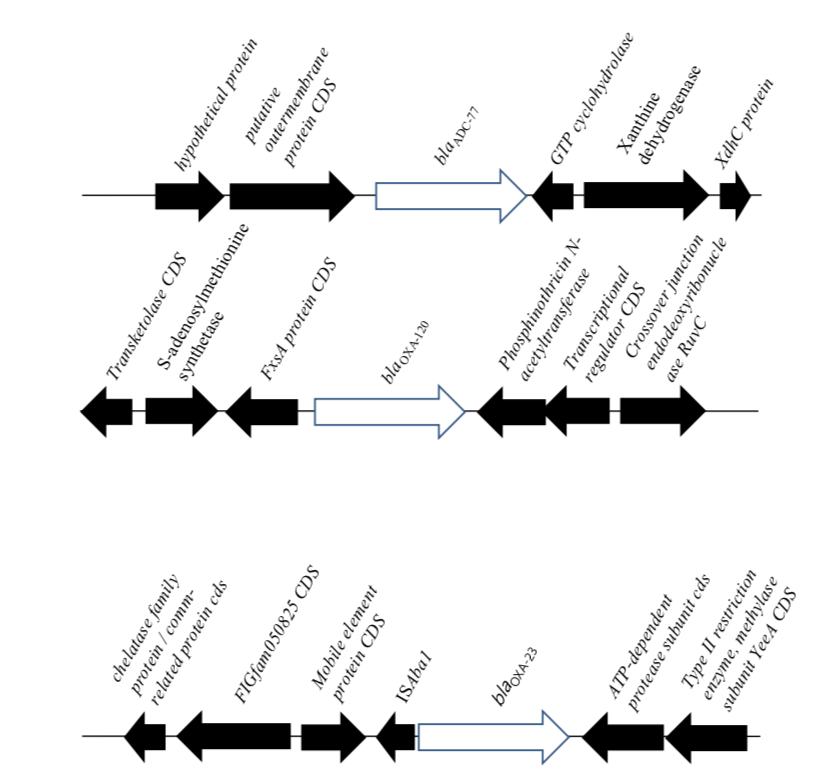


Figure S5-A: *A. baumannii* YMC2011/7/R812 expressing *bla*_OXA-120_, *bla*_OXA-23_and *bla*_ADC-77_.


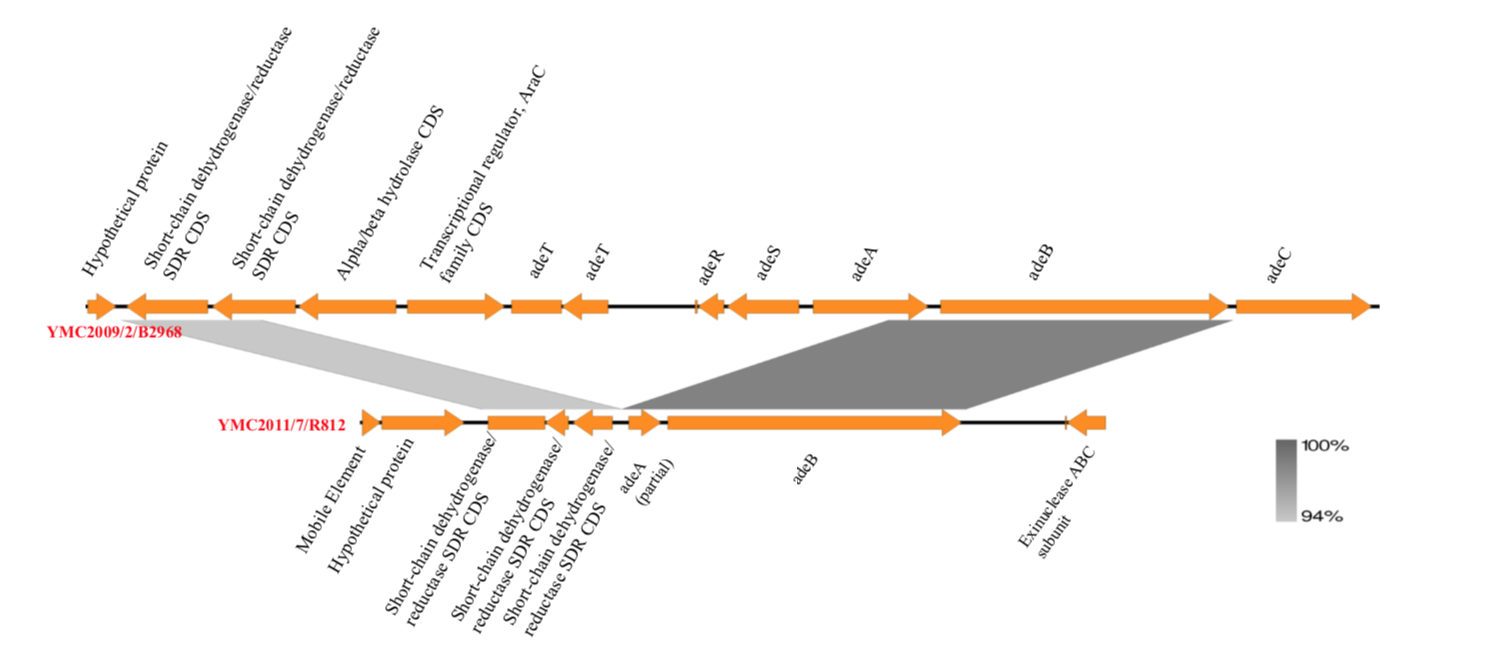


Figure S5-B: Genomic comparison of YMC2011/7/R812 with YMC2009/2/B2968 indicating the deletion of adeRS, two-component system regulating AdeABC expression system.


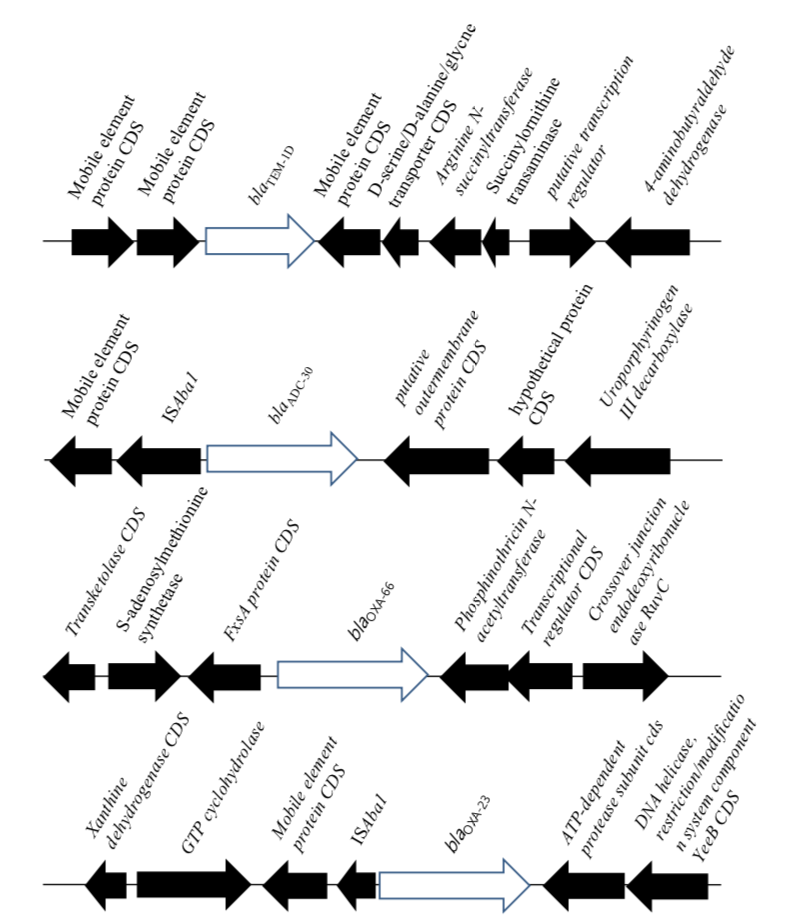


Figure S6: *A. baumannii* YMC2012/1/R79 expressing *bla*_TEM-1D_, *bla*_ADC-30,_ *bla*_OXA-23_ and *bla*_OXA-66._


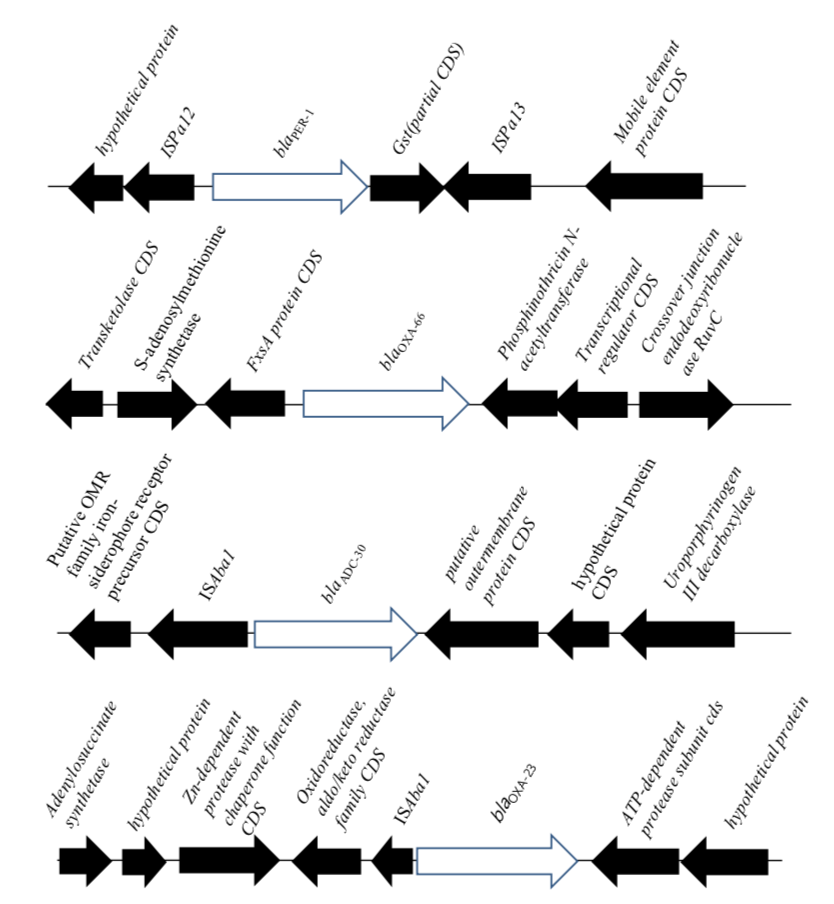


Figure S7: *A. Baumannii* YMC2011/2/C582 expressing *bla*_PER-1_*, bla*_OXA-66_, *bla*_OXA-23_ and *bla*_ADC-30._


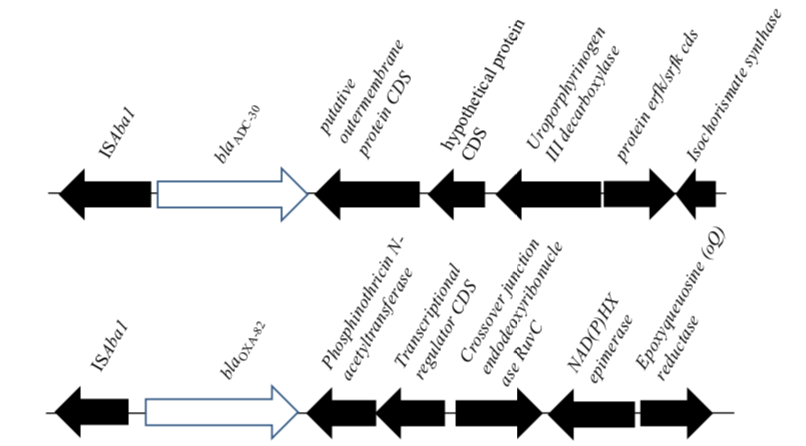


Figure S8: *A. Baumannii* YMC2012/9/R2209 expressing IS*Aba1-bla*_OXA-82_ and IS*Aba1*-*bla*_ADC-30._


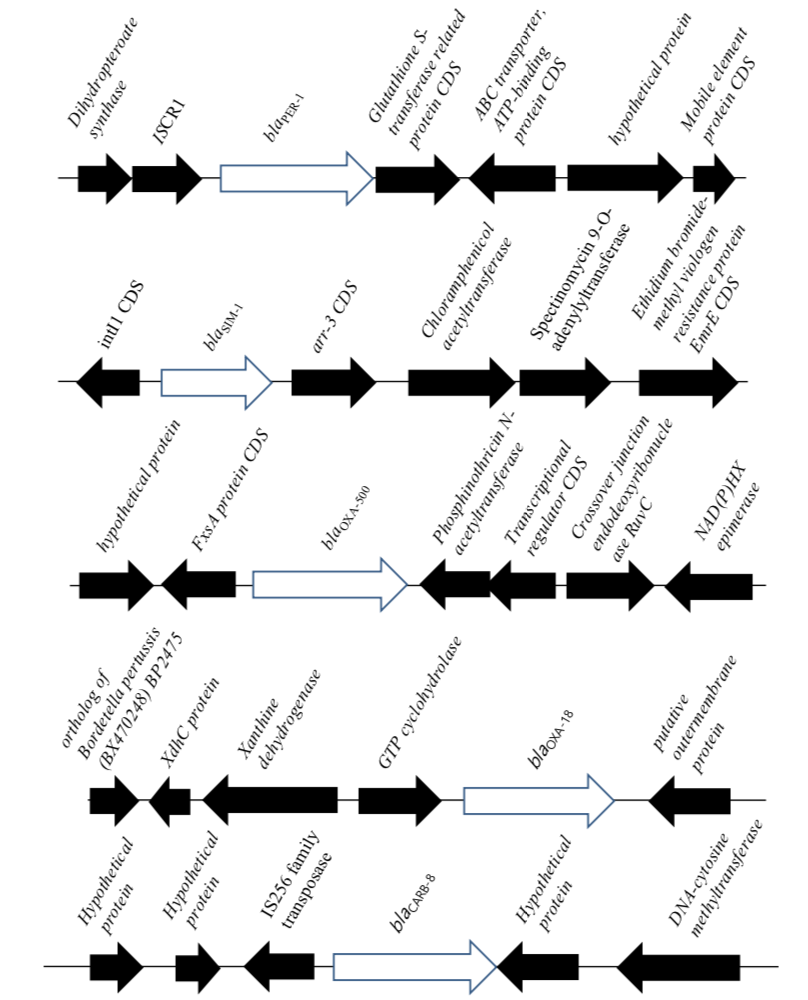


Figure S9: *A. pittii* YMC2013/3/R2081 expressing *bla*_PER-1_, *bla*_SIM-1_, *bla*_ADC-18_, *bla*_CARB-8_ and *bla*_OXA-500._


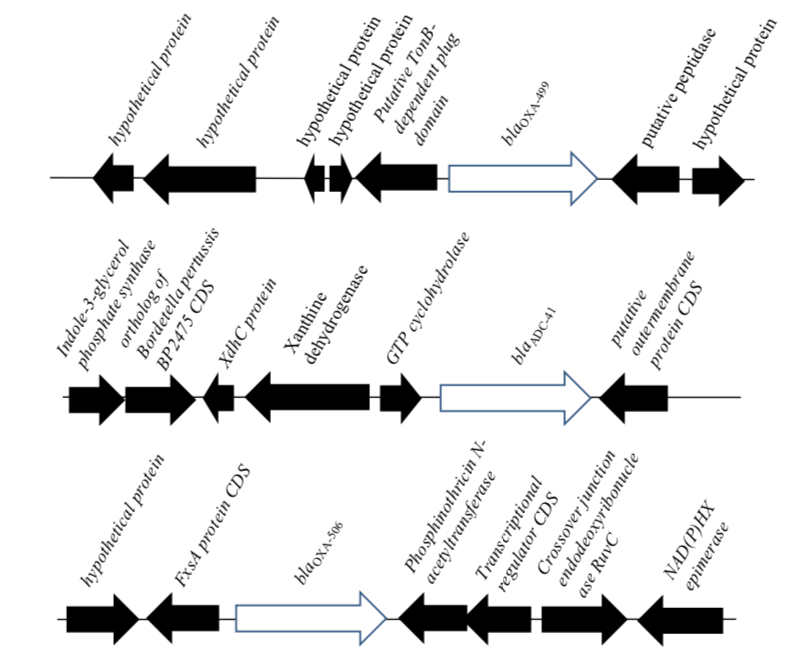


Figure S10: *A. pitti* YMC2010/8/T346 expressing bla_OXA-213-like_*, bla*_ADC-41_ and *bla*_OXA-499._


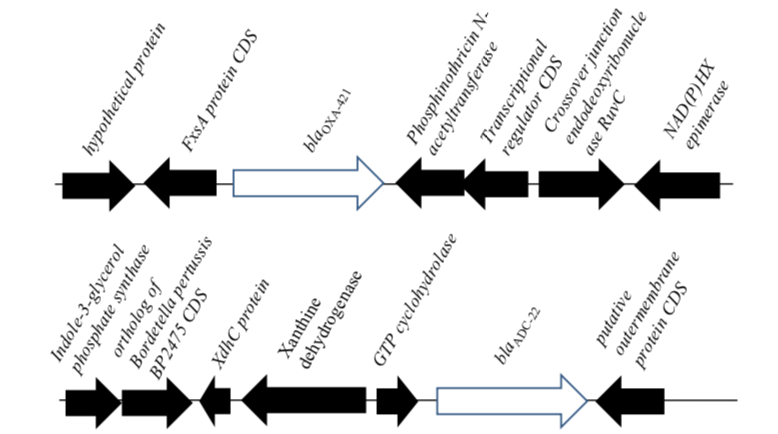


Figure S11: *A. pittii* YMC2009/2/B2968 expressing *bla*_OXA-213_ family and *bla*_ADC-22._


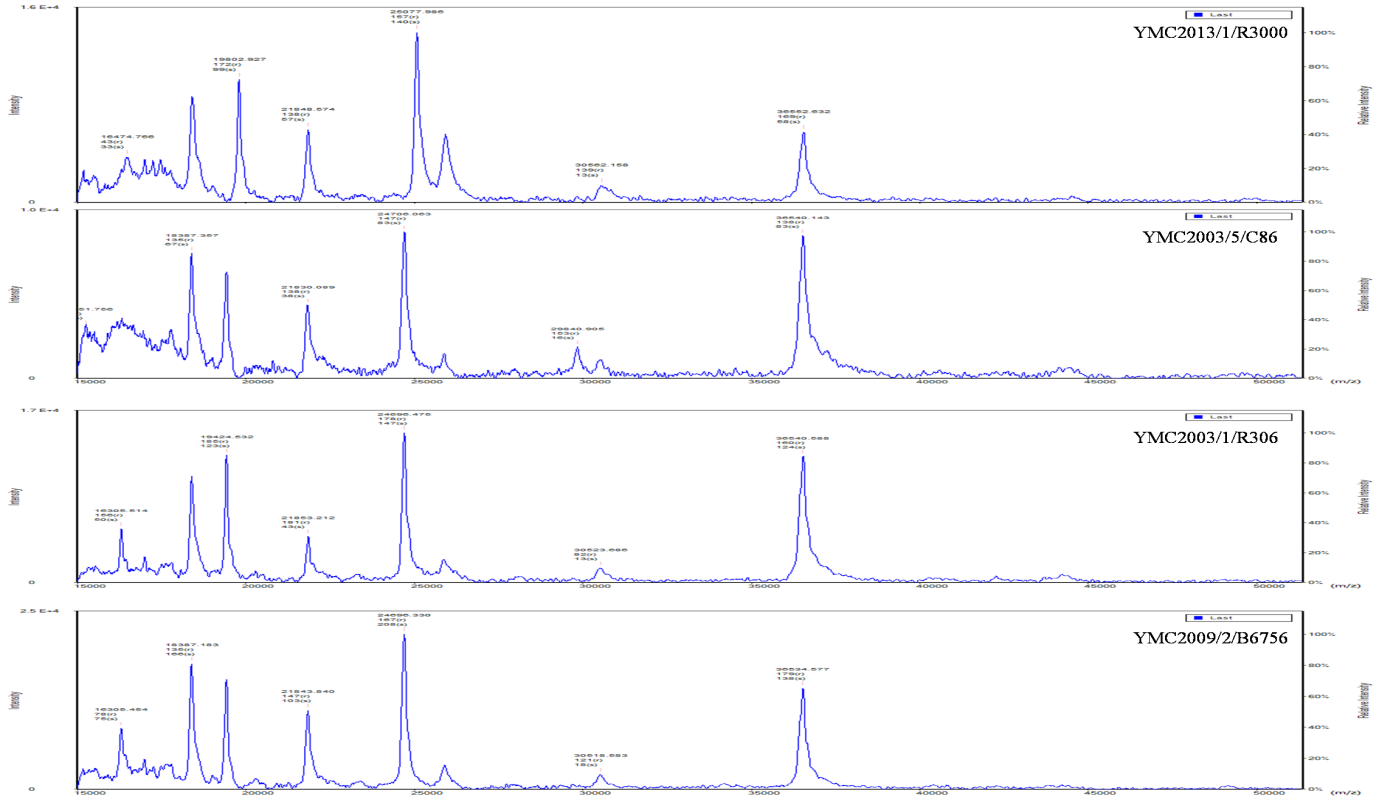


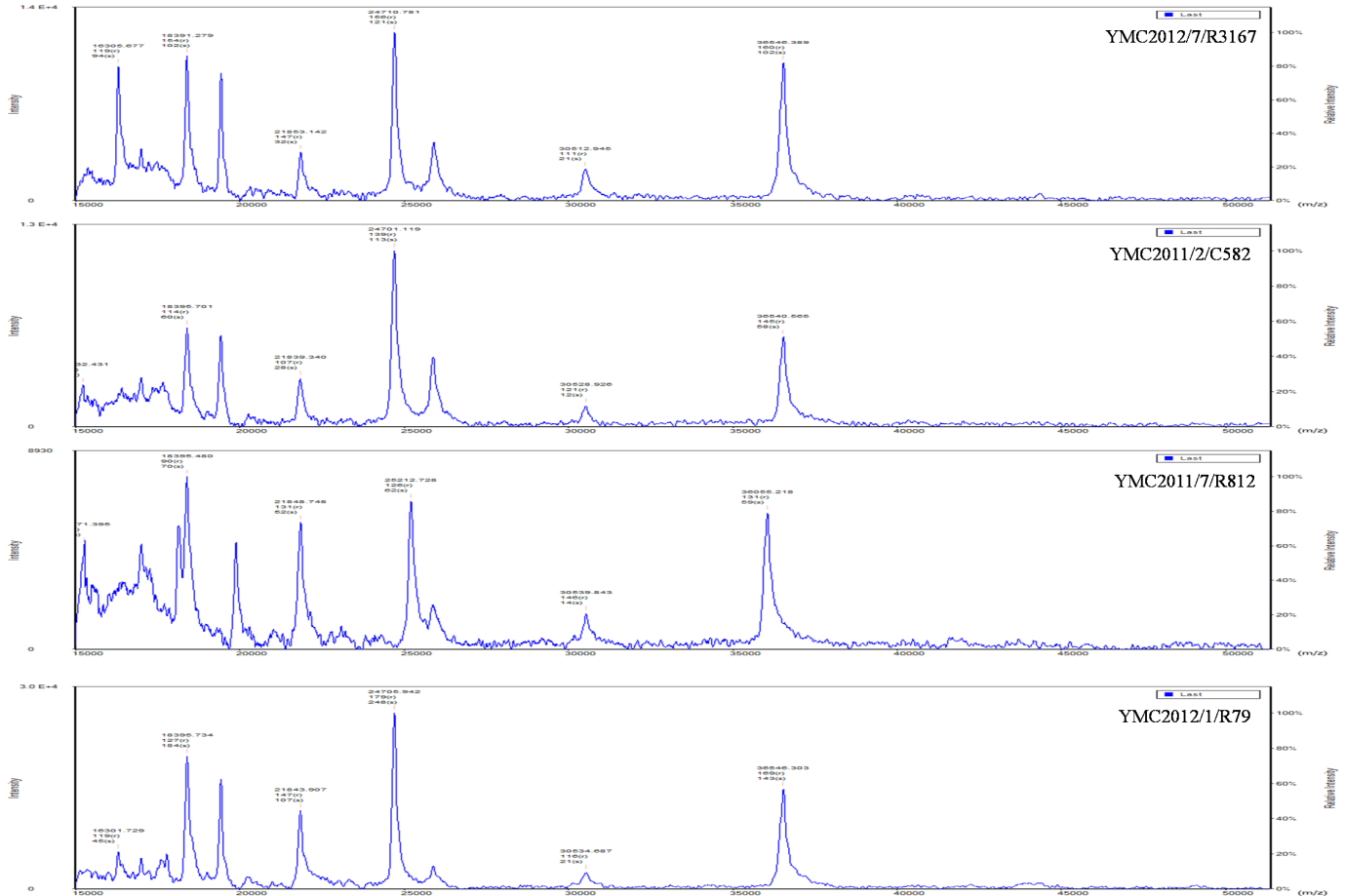


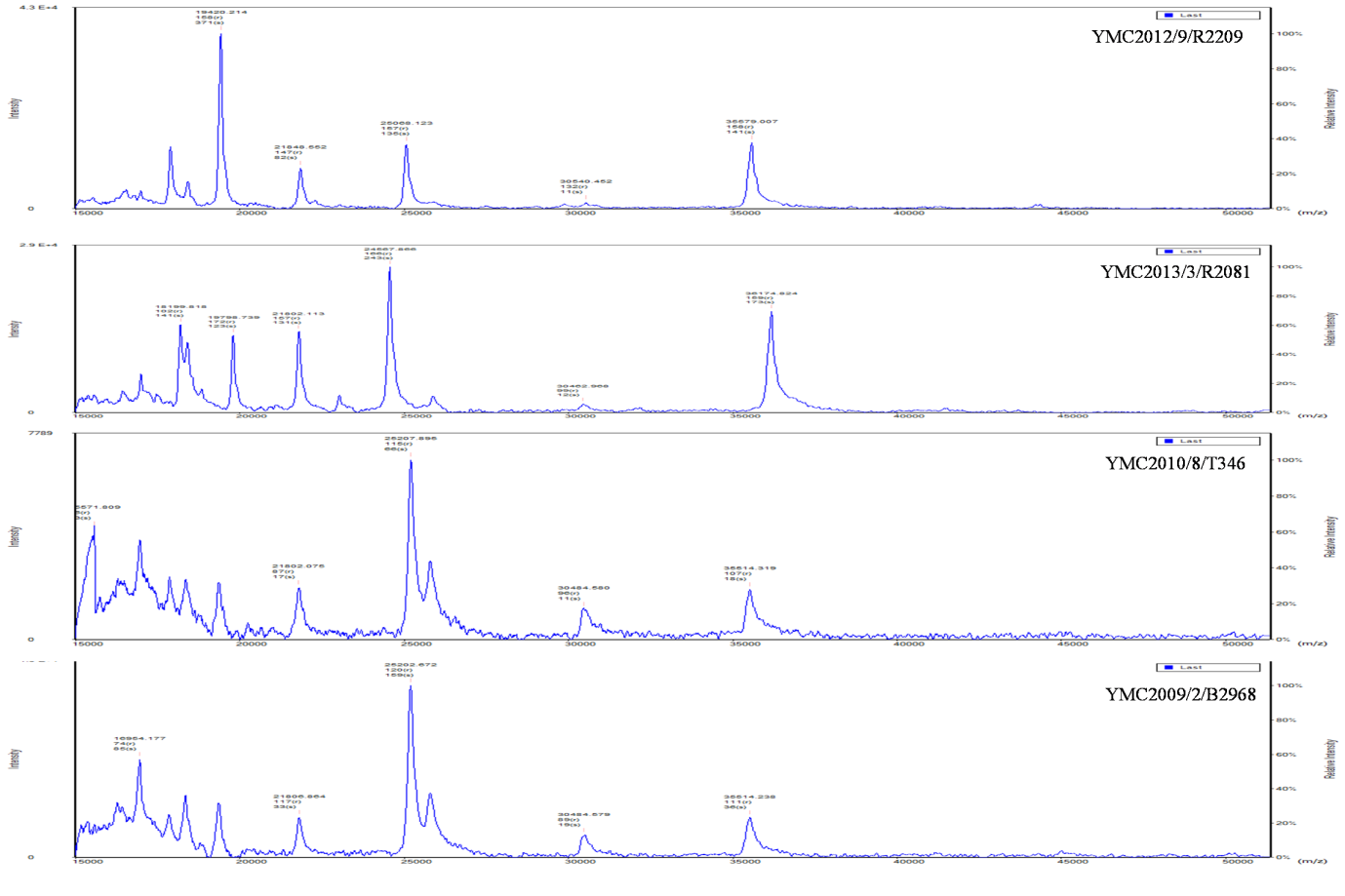


Figure S12: MALDI-TOF MS analysis of Acinetobacter isolates. x axis, mass per charge in Daltons (m/z, Da); y axis, absolute intensity of signal.

Figure S13: Expression of outer membrane proteins, *CarO, OprD* and *33-36 Kda omp.*

Table S9: Primer sequences used for qRT-PCR of *adeB, adeJ, adeG, BaeSR, CarO, OprD* and *33-36 Kda* genes.

|  |  | B6756 | R79 | R3167 | C582 | C86 | R812 | R2209 | R306 | R2081 | B2968 | T346 |
| --- | --- | --- | --- | --- | --- | --- | --- | --- | --- | --- | --- | --- |
| adeB For | CTTGCATTTACGTGTGGTGT | + | + | + | + | + |  |  |  |  |  |  |
| adeB Rev | GCTTTTCTACTGCACCCAAA | + | + | + | + | + |  |  |  |  |  |  |
| adeB For_2 | CTTGCATTTACCTGTGGTGT |  |  |  |  |  | + | + |  |  |  |  |
| adeB Rev_2 | GCTTTTCTACTGCACCCAGA |  |  |  |  |  | + | + | + |  |  |  |
| adeB For_3 | CTTGCGTTTACCTGTGGTGT |  |  |  |  |  |  |  | + |  |  |  |
| adeB Rev_3 | GTTTCTCTGCAGCTCCCAAC |  |  |  |  |  |  |  |  |  | + |  |
| adeB For_4 | TTAGCATTTACATGCGGAGT |  |  |  |  |  |  |  |  | + |  |  |
| adeB Rev_4 | ATTTATCAACAGTTCCCATA |  |  |  |  |  |  |  |  | + |  |  |
| adeB For_5 | CTTGCCTTTACCTGTGGTGT |  |  |  |  |  |  |  |  |  | + | + |
| adeB Rev_5 | GTTTCTCTGCGGCTCCCAAC |  |  |  |  |  |  |  |  |  |  | + |
| adeB Rev_6 | GCTTTTCTACTGCCCCCAGA |  |  |  |  |  |  |  |  |  |  |  |
| adeJ For | GGTCATTAATATCTTTGGC | + | + | + | + | + | + | + |  |  |  |  |
| adeJ Rev | GGTACGAATACCGCTGTCA | + | + | + | + | + | + | + | + |  |  |  |
| adeJ Rev_2 | GGTACGAATACCGCTGTTA |  |  |  |  |  |  |  |  | + | + |  |
| adeJ Rev_3 | GGTACGAATACGGCTGTTA |  |  |  |  |  |  |  |  |  |  | + |
| adeJ For_2 | TGTTATTAATATTTTTGGC |  |  |  |  |  |  |  | + |  |  |  |
| adeJ For_3 | TGTTATTAATATCTTTGGC |  |  |  |  |  |  |  |  | + | + | + |
| adeG For | TTCATCTAGCCAAGCAGAAG | + | + | + | + | + | + | + |  |  |  |  |
| adeG Rev | CCTGCTAATGGTAGGGTTAAG |  |  |  |  |  |  |  |  |  |  |  |
| adeG Rev_2 | CTGCTAATGGTAGGGTTAAG | + | + | + | + | + | + | + | + |  |  |  |
| adeG Rev_3 | CAGCTAAAGGCAAAGTCAGA |  |  |  |  |  |  |  |  | + |  |  |
| adeG Rev_4 | CTGCAAAAGGCATTGACCAA |  |  |  |  |  |  |  |  |  | + |  |
| adeG Rev_5 | CAGCTAAAGGCAAAGTTAGA |  |  |  |  |  |  |  |  |  |  | + |
| adeG For_2 | TTCATCTAGCCAAGCTGAGG |  |  |  |  |  |  |  | + |  |  |  |
| adeG For_3 | TTCATCTAGCCAAGCTGAAG |  |  |  |  |  |  |  |  | + |  |  |
| adeG For_4 | TTCATCTGGTCAGGCAACTG |  |  |  |  |  |  |  |  |  | + |  |
| baeSR_F | CGCGTAGTACAGGTGGAACA | + | + | + | + | + | + | + | + | + | + | + |
| baeSR_R2 | CGACTTCATCCTCAACCAACA | + | + | + | + | + | + | + |  |  |  |  |
| baeSR_R3 | CGACTTCATCTTCAACCAACA |  |  |  |  |  |  |  | + | + | + | + |
| carO_rt_F | TGCCGATGGTGTCAAAATTA |  |  |  |  |  |  |  |  |  |  |  |
| carO_rt_R | CGAATACGCCCCAGTTTTTA |  |  |  |  |  |  |  |  |  |  |  |
| carO_rt_R_2 | TACATATGGGTTGGCTTGCC |  |  |  |  |  | + |  |  |  | + | + |
| carO_rt_R_3 | TACATATGGGTTTGCTTGCC | + | + | + | + | + |  | + | + | + |  |  |
| carO_rt_F_2 | ACAGCTTTACTTGCTGCTGG | + | + | + | + | + | + | + | + |  |  |  |
| carO_rt_F_3 | ACAGCTTTATTCGCTGCTGG |  |  |  |  |  |  |  |  | + | + | + |
| oprD_rt_F | GTTTCGGTGTAGGCGTTGTT | + | + | + | + | + |  | + | + |  |  |  |
| oprD_rt_R | GGTCGTAAGCTGAACCATCG | + | + | + | + | + |  | + |  |  |  |  |
| oprD_rt_F_2 | GTTTCGGTGTGGGCATTGTT |  |  |  |  |  | + |  |  |  |  |  |
| oprD_rt_F_3 | GTTTCGGTGTTGGCGCTGTG |  |  |  |  |  |  |  |  | + | + | + |
| oprD_rt_R_2 | GATCGTAAGAATCACCGCAC |  |  |  |  |  | + |  |  |  |  |  |
| oprD_rt_R_3 | GATCGTAAGCTGAACCATCA |  |  |  |  |  |  |  | + |  |  |  |
| oprD_rt_R_4 | GATCATAAGCGGAGCCATCA |  |  |  |  |  |  |  |  | + | + | + |
| 33–36 kDa_rt_F | AATCGGTTTTGAAGCTGCTG | + | + | + | + | + | + | + | + |  |  |  |
| 33–36 kDa_rt_R | CCTACGAAAGTAGCGCCAAC |  |  |  |  |  |  |  |  |  |  |  |
| 33–36 kDa_rt_F_2 | AATTGGTTTTGAAGCTGCTG |  |  |  |  |  |  |  |  | + | + | + |
| 33–36 kDa_rt_R_2 | GTTTACGTTACCACCCCAAGCT | + | + | + | + | + | + | + | + | + | + | + |

Note: ' + ' indicates the specific primers used for PCR. Owing to the polymorphisms in the efflux pumps and porin genes, numerous primer sequences had to be designed.

Table S10: Experimental conditions used in RT-qPCR based on MIQE requirements.

| **Experimental design** | |
| --- | --- |
| Number of samples | 12 |
| Place of experiment performed | Department of Laboratory Medicine and Research Institute of Bacterial Resistance, Yonsei University College of Medicine |
| **Sample and Nucleic acid extraction** | |
| Type of sample | *Acinetobacter* spp. |
| Volume | 2 ml in LB broth with OD_600_ 0.7-0.8 |
| RNA extraction kit | RNeasy Mini Kit with RNAprotect Bacteria Reagent (Qiagen, Hilden, Germany) |
| Storage | Samples processed immediately |
| DNase treatment | On-column DNase I digestion for 15 min at RT (as described in the RNeasy Mini Kit) |
| Nucleic acid quantification | NanoDrop spectrophotometer (ND- 2000 Thermo scientific, USA) |
| Yield | 400-500 ng/ul |
| Purity | 260/280 ratio from 1.9 to 2.1, 260/230 ratio from 2.0 to 2.5 |
| RNA integrity | Analyzed by agarose gel electrophoresis |
| **Reverse Transcription** | |
| Complete reaction condition | Reaction performed as described by the M-MLV cDNA Synthesis Kit (Enzynomics, Korea) |
| Amount of RNA and reaction volume | 1 ug of RNA in 20ul of reaction volume. |
| Priming oligonucleotide | 50 μM random hexamers |
| Reverse transcriptase and concentration | M-MLV Reverse Transcriptase, 200U/ reaction |
| Temperature and time | 25°C for 10 min and then 42°C for 60 min, Inactivation at 95°C for 5 min |
| cDNA storage | -20°C and -80°C (for longer use) |
| **qPCR target information** | |
| Genes | *adeB, adeG, adeJ, baeSR, carO, 33-36kDa* *omp* and *oprD* genes |
| Amplicon length | 70-130bp |
| **qPCR oligonucleotides** | |
| Primer sequences | Please refer Table S9 |
| Manufacturer of oligonucleotides | Macrogen Inc, Korea |
| Purification method | MOPC |
| **qPCR protocol** | |
| complete reaction conditions | 95˚C for 3 min (1X) followed by 95˚C for 10 sec (40X) and 56˚C for 1 min(1X) |
| Reaction volume and amount of cDNA | 20-μl reaction volume containing 2 μl (100ng) of cDNA |
| qPCR kit | iQ SYBR Green Supermix (Bio-Rad, CA, USA) containing antibody-mediated hot-start iTaq DNA polymerase, dNTPs, MgCl2, SYBR® Green I dye, enhancers, stabilizers, and fluorescein |
| Manufacturer of tubes | MicroAmp Fast 8-Tube Strip, 0.1 mL (Cat: 4358293, Applied Biosystems) |
| Manufacturer of qPCR instrument | StepOne Real-Time PCR System (Life technologies, CA, USA) |
| **qPCR validation** | |
| Specificity | Dissociation curve was generated for each run |
| **Data Analysis** | |
| qPCR analysis program | StepOnePlus Software v2.2 (Life technologies, CA, USA) |
| Method of C_q_ determination and outlier identification | Calculated on Microsoft Excel using the method described by Livak and Schmittgen, 2001. (https://www.ncbi.nlm.nih.gov/pubmed/11846609) |
